# Overexpression of human virus surface glycoprotein precursors induces cytosolic unfolded protein response in *Saccharomyces cerevisiae*

**DOI:** 10.1186/1475-2859-10-37

**Published:** 2011-05-19

**Authors:** Evaldas Čiplys, Dhanraj Samuel, Mindaugas Juozapaitis, Kęstutis Sasnauskas, Rimantas Slibinskas

**Affiliations:** 1 Institute of Biotechnology, Vilnius University, V. Graiciuno 8, Vilnius, LT-02241, Lithuania; 2 Virus Reference Department, Centre for Infections, 61 Colindale Avenue, London, NW9 5HT, UK

## Abstract

**Background:**

The expression of human virus surface proteins, as well as other mammalian glycoproteins, is much more efficient in cells of higher eukaryotes rather than yeasts. The limitations to high-level expression of active viral surface glycoproteins in yeast are not well understood. To identify possible bottlenecks we performed a detailed study on overexpression of recombinant mumps hemagglutinin-neuraminidase (MuHN) and measles hemagglutinin (MeH) in yeast *Saccharomyces cerevisiae*, combining the analysis of recombinant proteins with a proteomic approach.

**Results:**

Overexpressed recombinant MuHN and MeH proteins were present in large aggregates, were inactive and totally insoluble under native conditions. Moreover, the majority of recombinant protein was found in immature form of non-glycosylated precursors. Fractionation of yeast lysates revealed that the core of viral surface protein aggregates consists of MuHN or MeH disulfide-linked multimers involving eukaryotic translation elongation factor 1A (eEF1A) and is closely associated with small heat shock proteins (sHsps) that can be removed only under denaturing conditions. Complexes of large Hsps seem to be bound to aggregate core peripherally as they can be easily removed at high salt concentrations. Proteomic analysis revealed that the accumulation of unglycosylated viral protein precursors results in specific cytosolic unfolded protein response (UPR-Cyto) in yeast cells, characterized by different action and regulation of small Hsps versus large chaperones of Hsp70, Hsp90 and Hsp110 families. In contrast to most environmental stresses, in the response to synthesis of recombinant MuHN and MeH, only the large Hsps were upregulated whereas sHsps were not. Interestingly, the amount of eEF1A was also increased during this stress response.

**Conclusions:**

Inefficient translocation of MuHN and MeH precursors through ER membrane is a bottleneck for high-level expression in yeast. Overexpression of these recombinant proteins induces the UPR's cytosolic counterpart, the UPR-Cyto, which represent a subset of proteins involved in the heat-shock response. The involvement of eEF1A may explain the mechanism by which only large chaperones, but not small Hsps are upregulated during this stress response. Our study highlights important differences between viral surface protein expression in yeast and mammalian cells at the first stage of secretory pathway.

## Background

Heterologous overexpression of proteins is connected with different stress reactions of the host cells and it can largely influence the productivity of an expression system [for review: [1]]. One of the main bottlenecks in recombinant protein production is the inability of the foreign polypeptides to reach their native conformation in heterologous host cells, which usually results in their prevalence in the insoluble cell fraction [2]. Especially complicated is the expression of functional eukaryotic membrane proteins which often suffered from low expression levels, instability of proteins and/or degradation by the host's proteolytic machinery [3]. The presence of misfolded or folding-reluctant protein species causes considerable stress in host cells. The characterization of such adverse conditions and the elicited cell responses have permitted a better understanding of the physiology and molecular biology of conformational stress [reviewed in [2]]. However, well-documented stress reactions in recombinant protein producing yeasts are limited mostly to unfolded protein response (UPR) in endoplasmic reticulum (ER) [2,3] and there is a lack of knowledge concerning the impact of other stress responses on heterologous membrane protein expression. Only recently two additional different stress responses induced by misfolded membrane proteins with lesions in a membrane span or a cytosolic domain (called UPR-M/C), and by misfolded cytosolic proteins that do not enter the secretory pathway at all (called UPR-Cyto) have been preliminarily characterized in *Saccharomyces cerevisiae *[4,5]. In our study, the analysis of virus surface protein overexpression revealed a stress response virtually indistinguishable from UPR-Cyto.

Viral glycoproteins in animal cells are synthesized by polysomes bound to the ER. The polypeptide precursors are usually transported through ER membrane cotranslationally and depend on signal recognition particle (SRP). Co-translational translocation places them into the ER lumen, where they fold and assemble into oligomeres, before being transported to the budding compartment [rev. [6-8]]. Several lines of evidence indicate that viral glycoproteins are not processed normally in yeast cells, resulting in abnormal folding and aggregate formation and the success observed in the case of hepatitis B surface antigen, HBsAg was an exception rather than the rule [9-11]. The expression of most other human virus surface glycoproteins is much more efficient in cells of higher eukaryotes rather than yeasts. Failure of yeast to produce active human virus surface glycoproteins indicates principal difference between yeast and mammalian cell secretion pathways. There are some reports where possible limitations of yeast expression systems to produce viral surface proteins are described including rather low expression level [12,13], formation of insoluble multimers [9,10] and inactivity due to hyperglycosylation [14]. However, earlier studies were focused only on the analysis of recombinant products, whereas the molecular processes that influence synthesis of these complex transmembrane proteins in yeast cells were not examined. Consequently, it remained unclear why human virus glycoprotein precursors fail to maturate in yeast cell, which cellular proteins and/or processes are involved and how these differ from mammalian cell secretion pathway. Elucidation of the reasons for these differences would provide important data concerning evolution of molecular mechanisms in eukaryotic cells and may help to improve yeast expression systems. A good example is the humanization of *N*-glycosylation pathway in yeast *Pichia pastoris*, that was based on an extensive knowledge about the main *N*-glycosylation pathways in yeasts and in humans [15]. Lately, system-wide analyses are emerging as powerful means of deciphering cellular bottlenecks during heterologous protein production. Omics technologies may provide new concepts to engineer microbial hosts for membrane protein production [reviewed in [3]].

The present study was undertaken to identify possible bottlenecks of recombinant viral glycoprotein production in yeast expression systems. We used several methods in parallel, including analysis of recombinant mumps hemagglutinin-neuraminidase (MuHN) and measles hemagglutinin (MeH) products synthesized in yeast as well as a proteomic study of yeast cell proteins in cells expressing or not expressing recombinant viral glycoprotein. Recombinant MeH, expressed in mammalian cells at high-level, is biologically active and correctly transported to the cell surface [16]. In contrast, MeH analogues produced in yeast cells were not processed normally, resulting in abnormal folding and formation of inactive intracellular aggregates. The same features were observed for recombinant MuHN protein. The results lead us to propose that inefficient synthesis of recombinant viral glycoproteins is determined by specific mechanisms at the early stages of yeast secretory pathway. Here we report a cytoplasmic unfolded protein response in yeast that appears as a marker for inefficient translocation of human virus surface glycoprotein precursors.

## Results

### Overexpression of MuHN and MeH in *S. cerevisiae *cells

**Figure 1:**
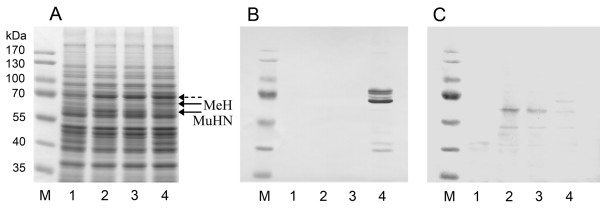
**Synthesis of recombinant MuHN and MeH causes overexpression of some cellular proteins in yeast**. (A-C) SDS-PAGE of whole cell lysates on a 10% polyacrylamide gel. All lysates were prepared from galactose-induced yeast cells of *S. cerevisiae *pep4 strain, transformed with empty vector (lane 1) or plasmids expressing MuHN (native sequence - lane 2 and with His6-tag - lane 3) or His6-tagged MeH (lane 4). Lane M - prestained protein ladder. (A) Coomassie blue-stained gel. Solid arrows indicate bands of recombinant MuHN (lanes 2 and 3) and MeH (lane 4) proteins. Dashed arrow points to ~70 kDa main band of yeast cellular proteins overexpressed in response to synthesis of MuHN and MeH (lanes 2-4). (B) Western blot using anti-His antibody. Expression of His-tagged recombinant MeH protein was confirmed by immunoblot analysis that revealed two main forms: major band of ~65 kDa and upper double band of ~75 kDa (lane 4, there are also a faint band just above 65 kDa and some degradation products visible). His-tagged recombinant MuHN protein did not react with anti-His antibody (lane 3). (C) Western blot using monoclonal antibody 782 to the native MuHN. Both native and His-tagged MuHN sequence variants were detected as ~60 kDa bands (lanes 2 and 3, respectively) corresponding to those shown in coomassie stained gel. Due to stronger reaction of Mab 782 with the native sequence MuHN variant the latter was chosen for further MuHN expression study. There was also some non-specific reaction and cross-reactivity with MeH observed.

The expression of MuHN and MeH in yeast cells has not been examined previously. The genes encoding the full-length mumps virus hemagglutinin-neuraminidase (MuHN) and measles virus hemagglutinin (MeH) were inserted into yeast expression vectors and expressed under the control of strong galactose-inducible promoters in *S. cerevisiae *cells. SDS-PAGE and Western blotting confirmed that recombinant MuHN and MeH proteins are expressed in yeast cells after induction with galactose (Figure 1), whereas no expression was detected after cultivation in growth medium with glucose (not shown). Expression of both MuHN and MeH inhibited yeast growth. Yeast harbouring empty vectors without viral genes doubled every 3 h in galactose media (YEPG), whereas the doubling time slowed to about 7 h for yeast expressing MuHN or MeH. During standard induction conditions [17,18] in YEPG medium a 1.4-fold decrease in wet biomass accumulation was observed in transformants carrying MuHN or MeH genes compared with control cells, harbouring empty expression vector pFGG3. In addition to yeast harbouring empty vectors used as controls, we also used *S. cerevisiae *strains expressing mumps and measles virus nucleocapsid proteins described earlier [17,18]. In contrast to MuHN and MeH, the expression of viral nucleocapsid proteins had no inhibitory effect on the yeast growth, despite the high-level of recombinant nucleoprotein expression. Thus, the synthesis of viral surface glycoproteins specifically inhibits yeast growth.

Analysis of whole cell lysates showed that synthesis of viral surface proteins causes overexpression of some cellular proteins, which were not recognized by monoclonal anti-His and anti-MuHN antibodies (Figure 1). Compared to the proteins observed after induction of yeast cells harbouring empty vectors, a ~70 kDa protein (Figure 1A, dashed arrow) is over expressed in induced yeast cells expressing MuHN and MeH. It is known, that expression of paramyxovirus HN glycoprotein in cells of higher eukaryotes stimulates synthesis of several cellular proteins, especially ER chaperone GRP78-BiP [19], however this effect is much less pronounced than the increase in amount of the 70 kDa protein in yeast. We performed proteomic analysis and overexpressed ~70 kDa cellular proteins were identified as cytosolic Hsp70 chaperones Ssa1/2p and Ssa4p, respectively (described below).

### Fractionation of yeast lysates and identification of eEF1A

**Figure 2:**
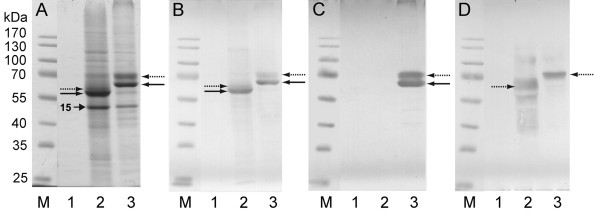
**SDS-PAGE of recombinant MuHN and MeH protein fractions insoluble in 8 M urea solution**. (A-D) The same samples were run on each gel: control sample from *S. cerevisiae *cells, transformed with empty vector was loaded on lane 1, whereas samples from *S. cerevisiae *expressing recombinant viral proteins were loaded on lanes 2 (MuHN) and 3 (MeH), respectively. (A) Coomassie blue-stained gel. Long solid arrows indicate major, whereas dotted arrows - minor forms of partially purified recombinant MuHN (lane 2) and MeH (lane 3). Short arrow points to ~50 kDa band analysed by MS directly from 1-D SDS-PAGE gel (the band number 15 is given according to the list of identified yeast proteins in Table 1). (B) Western blot using monoclonal antibody 782 to the native MuHN. In addition to the main band of ~60 kDa (solid arrow) the minor band (up to 65 kDa, dotted arrow) of MuHN was also distinguished (lane 2). Mab 782 cross-reacted with both major and minor bands of recombinant MeH (lane 3). (C) Western blot using anti-His antibody. Both ~65 kDa and ~75 kDa forms of MeH protein (indicated by solid and dotted arrows, respectively) were insoluble under mild denaturing conditions in 8 M urea solution (lane 3). (D) Western blot using Concanavalin A. The major forms of recombinant MuHN (~60 kDa) and MeH (~65 kDa) appeared to be non-glycosylated as they did not react with Concanavalin A (white band areas below dotted arrows in lanes 2 and 3, respectively). Only heterogeneous band of minor MeH form (~75 kDa) contained N-glycosylated protein reacting with Concanavalin A (lane 3, dotted arrow), similar result was observed in the case of MuHN minor form (lane 2).

Initially we attempted to purify recombinant viral surface proteins from yeast by Ni-NTA affinity chromatography, which allowed the efficient and rapid purification of biologically active histidine-tagged MeH from mammalian cells [16]. However, this procedure was unsuccessful under both native and denaturing conditions due to the extreme insolubility of yeast-expressed MuHN and MeH. Fractionation of yeast lysates demonstrated that *S. cerevisiae *synthesized MuHN and MeH proteins are totally insoluble under native conditions (i.e insoluble in the presense of non-ionic detergent and non-ionic detergent containing 1M NaCl, see Methods). Moreover, these proteins were insoluble even in 8 M urea (Figure 2). Recombinant MuHN and MeH were only solubilized under strong denaturing conditions in the presence of a reducing agent (described below). This indicates that MuHN and MeH were present in the insoluble multimeric forms due to intermolecular sulfhydryl bonds. Moreover, yeast-expressed MuHN did not react with monoclonal antibodies against native MuHN in dot blots, whereas recombinant MeH failed to react with measles positive human sera in ELISA. These results suggest that MuHN and MeH molecules were not processed normally in yeast cells, resulting in abnormal folding and disulfide-linked multimer formation. Similar observations were reported for rabies, vesicular stomatitis and Sindbis virus glycoprotein analogues produced in yeast [9,10].

**Figure 3:**
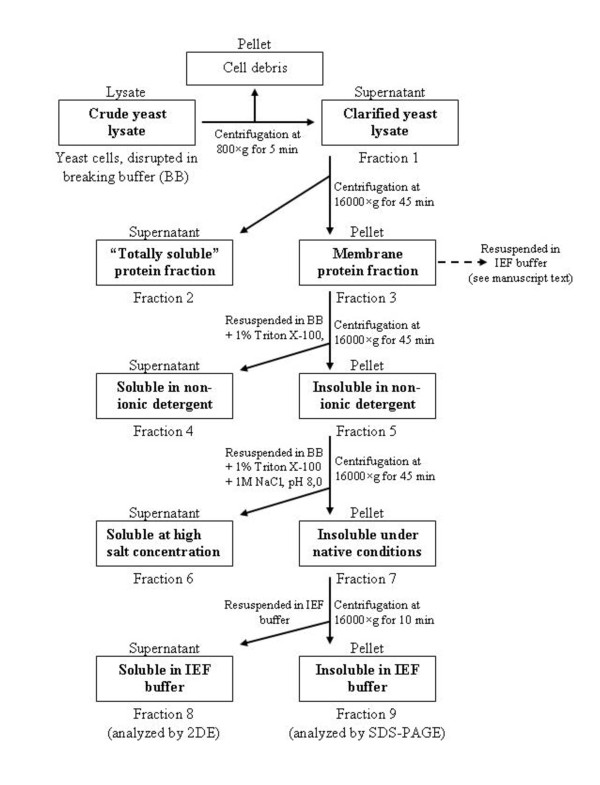
**A flow diagram for fractionation of yeast lysates**.

Recombinant MuHN and MeH were partially purified based on their insolubility in urea solution. After fractionation under native conditions, the pellets (Figure 3, fraction 7) containing insoluble proteins were resuspended in denaturing buffer B containing 8 M urea and soluble and insoluble proteins were separated by centrifugation. Virtually all yeast proteins were solubilized by this treatment in control samples, while the vast majority of viral protein remained insoluble. SDS-PAGE analysis of the insoluble fractions revealed that a group of proteins copurified with MuHN and MeH (Figure 2A). A major cellular protein associated with partially purified MuHN and MeH was detected as a ~50 kDa band (indicated by short arrow in Figure 2A, lanes 2 and 3, respectively). We were unable to separate this yeast protein by common 2D techniques (described below), therefore it was identified directly from 1D gel band by trypsin digestion and mass spectrometry. It appeared to be eukaryotic Translation Elongation Factor 1A (eEF1A or Tef1/2p according to *Saccharomyces *genome database; Table 1, protein number 15). eEF1A was removed from recombinant protein aggregates only by including a reducing agent (at least 60-100 mM 2-mercaptoethanol) which also solubilized MuHN and MeH. It suggests that eEF1A is involved in formation of complex with MuHN and MeH multimers.

**Table 1:** Identification of yeast proteins involved in recombinant MuHN and MeH expression.

Spot No.	**Name**^**a**^	**Fold Change**^**b**^	**ID Method**^**c**^	**Function, Process**^**d**^	Localization	**Fraction overexpressed**^**e**^
1,2 3	Ssa1/2 Ssa4	2.4 ± 0.2^f^	M, ESI ESI	Chaperone, Stress response Chaperone, Stress response	Cytoplasm, cell wall Cytoplasm, nucleus	All fractions except insoluble under denaturing conditions All fractions except insoluble under denaturing conditions
4	Kar2	3.8 ± 0.4	M	Chaperone, UPR	Endoplasmic reticulum	Soluble at high salt concentration
5	Sse1	2.3 ± 0.2	ESI	Co-chaperone, Stress response	Cytoplasm	Soluble at high salt concentration
6	Hsc82	2.1 ± 0.3	M, ESI	Chaperone, Stress response	Cytoplasm, mitochondrion	Soluble at high salt concentration
7	Eno2	1.5 ± 0.2	M	Lyase, Glycolysis	Cytoplasm, vacuole	Totally soluble
8	Sgt2	1.6 ± 0.2	ESI	Co-chaperone, Response to heat	Cytoplasm	Soluble in non-ionic detergent
9	Sti1	1.6 ± 0.3	M	Co-chaperone, Stress response	Cytoplasm	Soluble at high salt concentration
10	Hsp104	2.6 ± 0.3	ESI	Chaperone, Stress response	Cytoplasm, nucleus	Soluble at high salt concentration
11	Hsp26	0.7 ± 0.1	M	Chaperone, Stress response	Cytoplasm, nucleus	Insoluble under native conditions
12	Hsp42	ND^g^	M	Chaperone, Stress response	Cytoplasm, cytoskeleton	Insoluble under native conditions
13	Bgl2	ND^g^	M	Glycosidase, cell wall organization	Cell wall	Insoluble under native conditions
14	Pep4	ND^g^	M	Protease, respon- se to starvation	Vacuole, mitochondrion	Insoluble under native conditions
15	Tef2	NA^g^	ESI	Elongation factor, Translation	Cytoskeleton, Ribosome	Insoluble under denaturing conditions

^a^Accepted name from the *Saccharomyces *genome database (SGD) and YPD. Spots 1 and 2 represent mixtures of similar proteins Ssa1 and Ssa2 (97% identity) at an unknown ratio.

^b^Fold change represents ratio between the relative protein amounts (%Vol) in whole cell lysates of the MuHN/MeH expressing and control cells, respectively, ±SD.

^c^Protein identification method abbreviations: M, MALDI-fingerprint; ESI, nLC-ESI-MS/MS analysis.

^d^Molecular function, biological process and localization are noted according to UniProtKB and SGD.

^e^Lysates were fractionated into different fractions based on protein solubility under various conditions as described in Methods and shown in Figure 3. Fractions with the strongest overexpression of identified yeast proteins in MuHN and/or MeH variants compared to control samples are indicated.

^f^Three closely packed spots 1, 2 and 3 were merged and the fold change calculated for one common Ssa spot.

^g^ND - not determined; NA - not assayed.

### Glycosylation of recombinant MuHN and MeH in yeast

Western blotting of whole cell lysates using anti-His antibody revealed major and minor forms of recombinant MeH protein (Figure 1B, lane 4). Densitometric analysis of the protein bands detected with anti-His antibody showed that the major band constitutes ~80% of total MeH amount in *S. cerevisiae *AH22 cells. Major and minor forms of MuHN were also distinguished in partially purified samples using monoclonal antibody against native MuHN protein (lanes 2 in Figure 2A and 2B). The vast majority of recombinant MuHN protein (~90%) was present in the major band.

**Figure 4:**
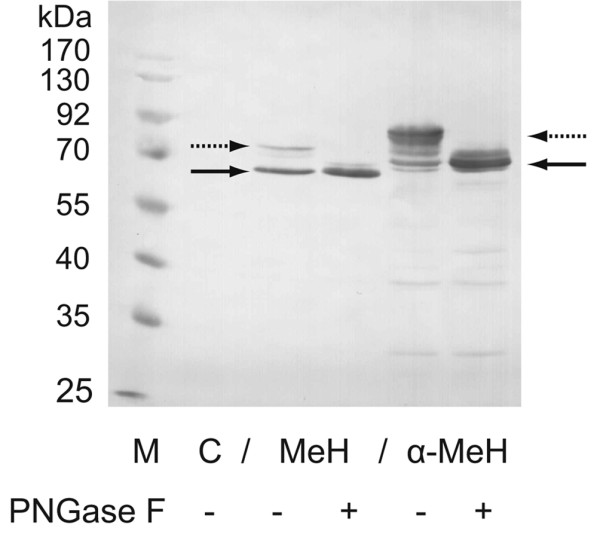
**PNGase F treatment of *S. cerevisiae *whole cell lysates followed by Western blotting using anti-His antibody**. Lysates were prepared from galactose-induced yeast cells, transformed with either an empty vector (lane C) or plasmids expressing the full sequence MeH variant (pFGG3-MeH) and chimeric α-MeH protein with α-factor signal sequence instead of native N-terminal TM domain (pFGG3-alpha-MeH). Lysates with (+) or without (-) PNGase F treatment are indicated below blot lanes. Solid arrows indicate unglycosylated MeH polypeptides, dotted arrows - glycosylated MeH forms.

The molecular weight, estimated from SDS-PAGE, of the major forms of recombinant MuHN and MeH proteins were ~60 kDa and ~65 kDa, respectively (Figures 1 and 2), approximately corresponding to molecular weight estimates of the MuHN and MeH polypeptides from the nucleic acid sequences. It is not consistent with molecular weights of native MuHN and MeH glycoproteins, synthesized in mammalian cells. Due to N-glycosylation the molecular weight of these proteins increases by ~10 kDa. Glycosylated MeH protein usually constitutes two bands of 74-78 kDa [20], and MuHN glycosylated forms of 74-76 kDa are observed [21]. In this study we assessed the glycosylation of yeast synthesized MeH and MuHN by Western blotting of partially purified proteins using a lectin conjugated to horseradish peroxidase (Figure 2D). For both MuHN and MeH, the minor component of the purified proteins was stained with concanavalin A, whereas the major forms of yeast-expressed MeH and MuHN correspond to unglycosylated polypeptides remained unstained (Figure 2D). Digestion of MeH with PNGase F in whole cell lysate confirmed that the major protein band of ~65 kDa represents unglycosylated form and only minor bands of ~75 kDa contain the N-glycosylated protein (Figure 4, MeH lanes).

### MeH expression in *P. pastoris*, the effects of gene dosage and the use of yeast secretion signal for viral glycoprotein expression

**Figure 5:**
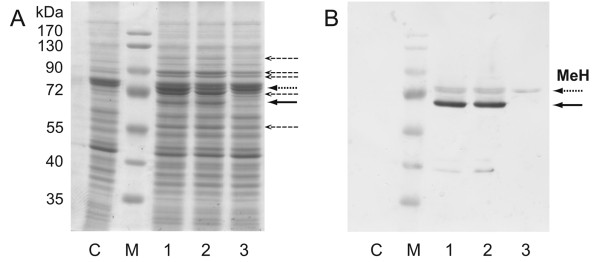
**Expression of His-tagged MeH in *P. pastoris *transformants induced with methanol**. (A) SDS-PAGE of whole cell lysates from *P. pastoris *transformants carrying empty vector or multicopy MeH expression cassetes. (B) Western blotting of the same samples using anti-tetraHis antibody. M- prestained protein marker, C- Control (pPIC3.5K Mut^S^), 1,2,3- multicopy pPIC3.5K-MeH transformants for intracellular expression of MeH, resistant to various concentrations of antibiotic G418. Solid arrows indicate unglycosylated MeH polypeptide precursor, dotted arrows point to glycosylated MeH form and dashed arrows show cellular proteins, upregulated in response to MeH expression.

**Figure 6:**
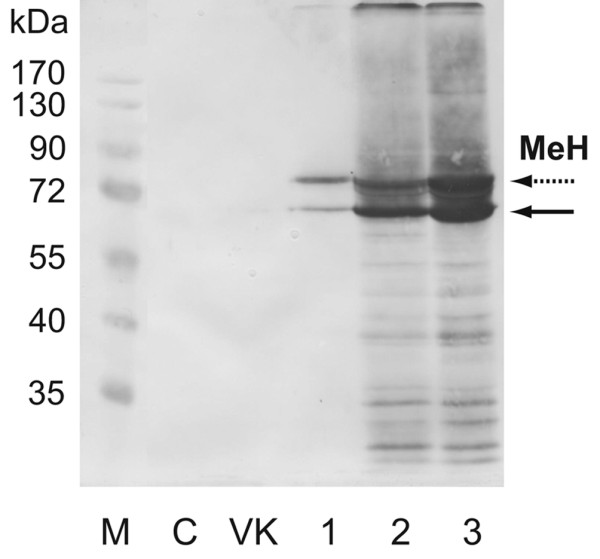
**Analysis of MeH in insoluble protein fractions from *P. pastoris *by Western blotting using anti-His antibody**. The fractions insoluble in 1% TritonX-100 and 1M NaCl were obtained from *P. pastoris *transformants using the same method as described in Methods for *S. cerevisiae *samples (correspond to fraction 7 in Figure 3). VK indicates the sample obtained from *P. pastoris *transformant carrying one copy of pPIC3.5K-MeH expression cassete. Other markings are the same as in Figure 5 (dotted arrow indicates glycosylated MeH form, whereas solid arrow - unglycosylated MeH precursor).

**Figure 7:**
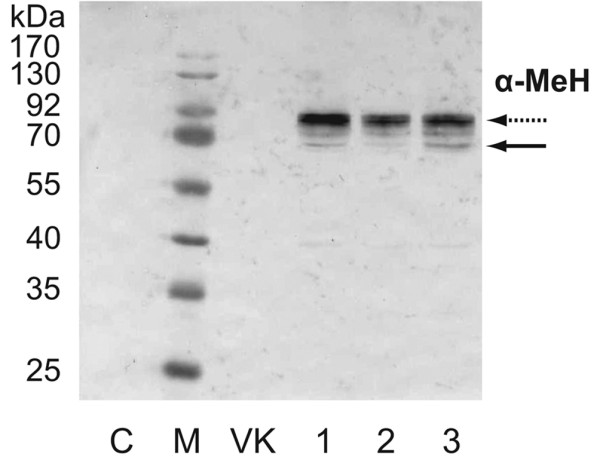
**Analysis of the expression of α-MeH chimeric protein containing *S. cerevisiae *α-factor signal sequence**. Whole cell lysates of methanol induced *P. pastoris *cells (Mut^+ ^phenotype) expressing α-MeH chimeric protein containing *S. cerevisiae *α-factor signal sequence instead of native TM anchor domain were resolved by SDS-PAGE, blotted onto nitrocellulose membrane and analysed by Western blotting using anti-His antibody. M- protein marker, C- control (pPIC9K Mut^+^), VK- one-copy pPIC9K-MeH transformant, 1,2,3- multicopy pPIC9K-MeH transformants. Solid arrow indicates unglycosylated MeH polypeptides, dotted arrow - glycosylated MeH forms.

**Figure 8:**
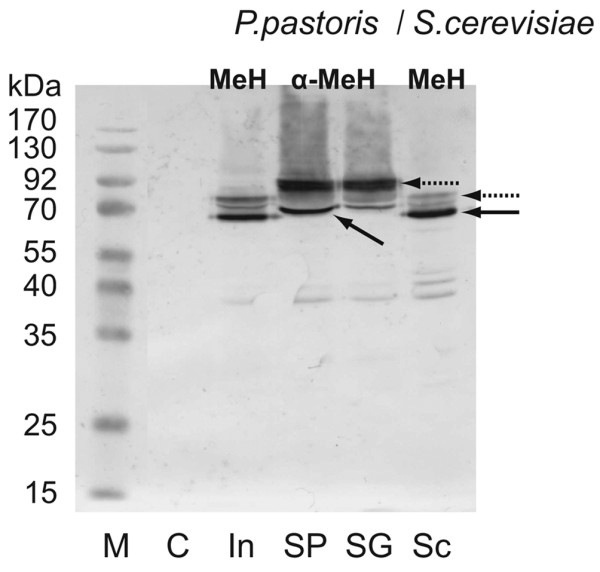
**Analysis of MeH expression in whole cell lysates from *P. pastoris *and *S. cerevisiae***. Whole cell lysates from *P. pastoris *(C, In, SP and SG) and *S. cerevisiae *(Sc) were analysed by Western blotting using anti-His antibody. M- marker, C- control, In- multicopy pPIC3.5K-MeH transformant for intracellular expression of full sequence MeH protein, SP- multicopy pPIC9K-MeH transformant for secreted expression of chimeric α-MeH protein with α-factor signal sequence (overexpressing both polypeptide precursor and glycosylated MeH forms), SG- another multicopy pPIC9K-MeH transformant for secreted expression of chimeric α-MeH, overexpressing glycosylated MeH form, Sc- pFGG3-MeH transformant of *S. cerevisiae *AH22 strain overexpressing full sequence MeH protein after induction with galactose. Solid arrows indicate unglycosylated MeH polypeptides, whereas dotted arrows - glycosylated MeH forms.

Our primary goal was to successfully express viral glycoproteins, therefore we have checked the expression of MeH by lowering gene dosage (in *P. pastoris*) and also expressed both MuHN and MeH using moderate promoters (in *S. cerevisiae*). However, this manipulation did not enhance translocation rate and solubility of recombinant protein. A comprenhensive study was performed on MeH expression in *P. pastoris *system (illustrated by Figures 5, 6, 7 and 8) that gives much more options for such experiments than *S. cerevisiae*. We have cloned the same His-tagged *MeH *gene into *P. pastoris *vector pPIC3.5K under control of *AOX1 *promoter and electroporated into *P. pastoris *strain GS115 with subsequent selection of multicopy transformants, resistant to various concentrations of antibiotic G418. The expression analysis of transformants with increasing expression level of MeH revealed the following results. The expression of MeH in transformants with one copy of integrated *MeH *gene is too low to be detected by Western blot. A range of MeH expression level is detected in multicopy pPIC3.5K-MeH transformants, resistant to various G418 concentrations. When MeH is expressed at the low level, all recombinant product is translocated into ER and glycosylated (Figure 5B, lane 3). Increasing the expression level, the protein begins to accumulate in the cytosol in the form of unglycosylated precursors. Further increasing gene dosage results in the accumulation of large amounts of unglycosylated MeH precursors (Figure 5B, lanes 1-2), similar to that observed for overexpressed MeH and MuHN in *S. cerevisiae*. Unfortunately, lowering the expression level has no effect on properties of recombinant MeH protein. All synthesized protein was detected in the insoluble fractions in transformants with both high and low level expression of MeH (Figure 6, lanes 1-3; this fraction was derived from *P. pastoris *by the same method as from *S. cerevisiae *and corresponds to fraction 7 in Figure 3), whereas MeH was not detected in soluble protein fractions. The same result was obtained in *S. cerevisiae *using moderate promoter for MeH and MuHN expression (not shown). Less amount of recombinant protein was achieved (compared to that using strong promoters), however it was insoluble in similar aggregates as described above. Therefore, the only effect of using a moderate and not a strong promoters was a decrease in the expression level of MeH with a less pronounced stress response (Figure 5A, lane 3 in comparison to lanes 1-2), whereas the recombinant product remained insoluble and inactive.

We have also used *S. cerevisiae *MFalpha1 (α-factor) signal sequence for MeH expression. The 5' part of *MeH *gene encoding N-terminal transmembrane (TM) anchor domain was removed and the rest of the gene was in frame fused with 269 bp fragment encoding the α-factor signal sequence by cloning into *P. pastoris *vector pPIC9K. We have expressed this gene encoding chimeric α-MeH protein in both *P. pastoris *and *S. cerevisiae*. Indeed, the α-factor signal sequence was much more effective for translocation of MeH precursors. When we used the same powerful pFGG3 expression vector (as in the case of overexpression of the native sequence MeH variant, described above) for α-MeH expression in *S. cerevisiae*, a majority of recombinant MeH protein was translocated into ER and glycosylated (Figure 4, α-MeH lanes). However, we did not achieve active recombinant protein by this manipulation and observed that MeH with the α-factor signal sequence induces different physiological response than full-length native sequence MeH protein (to be reported elsewhere). Briefly, this chimeric protein aggregated in the ER lumen and was not secreted in the culture media. The same results were obtained in *P. pastoris*. The gene dosage effect was similar as in the case of native MeH sequence variant. When the expression level exceeded the limit of successful translocation, the unglycosylated α-MeH precursors began to accumulate in the cytoplasm (Figure 7, lanes 1 and 3, compared to lane 2; Figure 8, lane SP compared to lane SG). Similarly as in the case of native sequence MeH variant expression, recombinant α-MeH protein from both yeast genera was insoluble and was not recognized by measles positive human sera. Therefore, the standard approaches to achieve successful expression of viral protein did not help in this case.

It is possible to evaluate *P. pastoris *versus *S. cerevisiae *for viral glycoprotein expression, because we have tried to express MuHN and MeH in both systems. As it is described above, the expression of MeH gave similar results in both expression systems. The lysates obtained from both yeast genera overexpressing the native sequence MeH variant are also shown on the same blot in Figure 8 (lanes In and Sc, respectively). Glycosylation of MeH protein variants expressed in *P. pastoris *was assessed by the same methods as for *S. cerevisiae*, including probing with Concanavalin A and treatment with PNGase F (PNGase F digestion of *S. cerevisiae *expressed MeH and α-MeH is shown in Figure 4), similar results were obtained with *P. pastoris *system. The similar forms of MeH protein were obtained in both yeast genera. Of course, the expression of MeH in *P. pastoris *is much more informative, because we could achieve a range of MeH expression levels. Interestingly, the MuHN protein (both native sequence and α-MuHN variants) was not expressed in *P. pastoris *at all. We suggest that it was due to premature termination of the transcription, because *MuHN *gene possesses specific AT rich sequences (nt 417-426 and nt 1669-1679). Similar sequences were shown to act as a premature polyadenylation sites in *P. pastoris*, resulting in the production of truncated mRNA [14]. This is a species-specific phenomenon of *P. pastoris*; the differences in mRNA 3'-end formation between the *P. pastoris *and *S. cerevisiae *have been reported earlier [14,22]. It should be possible to render *MuHN *gene expression in *P. pastoris *by increasing GC content in those AT rich sites, as was done in the case of genes encoding HIV-1 envelope glycoprotein gp120 [14] and SARS CoV glycoprotein S1 [23]. However, we saw no reason to do this, because we could express the native variant of *MuHN *gene in *S. cerevisiae *(described in previous sections). As regarding MeH expression study in *P. pastoris*, we have also carried out similar two-dimensional (2D) electrophoresis experiments as in *S. cerevisiae *(presented below). However, at that time the full sequence of *P. pastoris *genome was not publicly available and this was an obstacle to identification of cellular proteins by mass spectrometry (MS). Due to this we chose *S. cerevisiae *as model system to further study viral surface glycoprotein expression in this work.

### Proteomic analysis reveals different action and regulation of small heat shock proteins versus large Hsps

**Figure 9:**
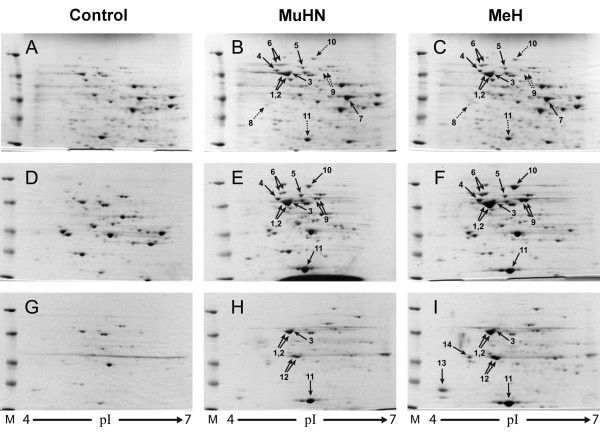
**Recombinant MuHN and MeH induce specific stress response in yeast cells**. (A-I) 2D gel electrophoresis of yeast proteins. Samples were taken from yeast cells of *S. cerevisiae *AH22 strain, expressing MuHN (central panel; B-H) or MeH (right panel; C-I) and from control AH22 cells, transformed with empty vector pFGG3 (left panel; A-G). At the top (A-C) whole cell lysates, in the middle (D-F) fractions of proteins, soluble at high salt concentration, and in the bottom panel (G-I) proteins, insoluble under native conditions are shown. Solid arrows in B and C indicate proteins, identified by MS directly from whole cell lysates, whereas dotted arrows point to the proteins, identified from soluble and insoluble fractions. Numbers of identified protein spots correspond to those, given in Table 1. Protein molecular mass markers (120, 66, 45, 35, and 25 kDa) were run simultaneously in the left lane (M) of each 2D gel.

Two-dimensional (2D) gel electrophoresis and matrix-assisted laser desorption/ionization-mass spectrum (MALDI-MS) fingerprinting with additional nanoscale liquid chromatography - electrospray ionization - tandem mass spectrometry (nanoLC-ESI-MS/MS) were used to separate and analyse the proteins (see Methods section). 2D gel electrophoresis of whole cell lysates from *S. cerevisiae *using a broad-range pH gradient (pH 3-10) showed that the vast majority of differently expressed protein spots are focused within a pI range of 4-7 and with an Mw ranging from 20 to 120 kDa. Consequently, we used a narrow range pH gradient (pH 4-7), which allowed us to resolve a major ~70 kDa band of overexpressed cellular proteins into three closely packed spots on 2D gels (Figure 9B, spots 1-3). They were identified by MS as cytosolic Hsp70 chaperones Ssa1/2p and Ssa4p, respectively. Seven more abundant proteins upregulated in response to MuHN and MeH synthesis were identified directly from 2D gels of *S. cerevisiae *whole cell lysates, whereas others were identified from fractions obtained after fractionation. We have also identified cellular proteins Adh1, Adh2, Eno1 and Pdi1, which according to relative protein amounts were repressed in protease deficient *S. cerevisiae pep4 *strain producing MuHN and MeH, however this effect was less evident in parental AH22 strain. It is unclear whether this repression shows true biological effect or results from variation among yeast strains. On the other hand, aforementioned cellular proteins were totally soluble and did not interact with MuHN and MeH, therefore their expression was not further explored. Only the proteins interacting with recombinant viral protein aggregates or exhibiting positive expression change are indicated in Table 1.

As indicated in Table 1, most of the identified proteins, overexpressed in response to synthesis of MuHN and MeH, are chaperones and co-chaperones involved in cellular stress responses. All of them, except the ER-resident chaperone Kar2p/BiP, are localized in the cytoplasm. Ssa1/2p, Ssa4p and Sse1p represent cytosolic Hsp70 family, Hsc82p is a member of Hsp90 family and Hsp104p belongs to Hsp110 family, respectively. Sti1p and Sgt2p are co-chaperones, which utilize tetratricopeptide repeats (TPR) to interact with identified Hsp70, Hsp90 and Hsp110 chaperones [24,25] and thereby coordinate and regulate the activities of the large Hsps in chaperone complexes [26,27]. Figure 9A-C and data presented in Table 1 demonstrate that MuHN and MeH induce cytoplasmic stress, which upregulates the expression of large Hsps.

The interaction of cellular proteins with MuHN and MeH was studied after fractionation of yeast lysates using centrifugation and extraction. This was compared to cellular proteins obtained from induced control yeast cells harbouring empty vectors. A detailed procedure for fractionation of yeast lysates is described in Methods and a step-wise diagram is given in Figure 3. Fractionation of cell lysates revealed that Hsp70 chaperones Ssa1/2p and Ssa4p were similarly overexpressed in all soluble fractions, whereas other identified proteins were found in separate fractions (Table 1), showing different degrees of interactions with MuHN and MeH. Enolase 2 (Eno2p) was the only identified protein with increased expression that did not interact with MuHN or MeH aggregates and remained completely soluble. Treatment with Triton X-100 fully solubilized only co-chaperone Sgt2p (not shown), indicating hydrophobic interaction with protein aggregates, whereas large Hsps were solubilized from MuHN and MeH at high salt concentration (Figure 9E and 9F), suggesting ionic interactions. Surprisingly, in the latter fraction we detected a new protein (spot 11) interacting with both MuHN and MeH. Analysis of protein fractions insoluble under native conditions by 2D gel electrophoresis showed three major cellular components, exhibiting the strongest interaction with MuHN and MeH: a major protein spot 11, double spot 12 and spots 1-3 (Figure 9H and 9I). Spots 11 and 12 were identified as small heat shock proteins 26 and 42 (Hsp26 and Hsp42, respectively), whereas spots 1-3 corresponded to Hsp70 chaperones, identified from cell lysates. Small heat shock proteins (sHsps) also appeared to be involved in cellular stress response, however these proteins displayed different interactions with MuHN and MeH. The vast majority of overexpressed large Hsps were solubilized under native conditions, whereas the majority of sHsps could only be removed from insoluble viral protein aggregates under denaturating conditions. This demonstrates the formation of irreversible protein aggregates containing sHsps, which can not be recovered *in vivo *and under native conditions *in vitro*. Moreover, in contrast to large Hsps, expression of Hsp26 was not upregulated, whereas Hsp42 expression was too low to be detected in whole cell lysates on 2D gels (Figure 9A-C and Table 1). These results demonstrate a specific cellular stress with different action and regulation of sHsps versus large Hsps in response to synthesis of MuHN and MeH. Finally, two additional proteins, involved only in MeH aggregates were identified as Bgl2 and Pep4 (spots 13 and 14, respectively).

### Analysis of viral protein aggregates involving eEF1A

After fractionation of *S. cerevisiae *cell lysates and extraction of insoluble fractions with Isoelectric focusing (IEF) sample running buffer, remaining insoluble pellets (Figure 3, fraction 9) was analysed by SDS-PAGE. It showed a pattern of insoluble proteins similar to that seen in Figure 2A. A major cellular protein interacting with insoluble MuHN and MeH was identified as eEF1A (described above). To investigate insoluble aggregates involving eEF1A in more detail we analyzed membrane protein fractions (Figure 3, fraction 3) that were not treated with non-ionic detergent. The pellets were dissolved in denaturing IEF buffer (including 7M urea/2M thiourea and 2% CHAPS) with or without reducing agent (75 mM DTT) and insoluble aggregates were separated by centrifugation. SDS-PAGE analysis of insoluble material after treatment with IEF buffer without reducing agent revealed similar composition of MuHN and MeH aggregates as shown in Figure 2A. It confirmed the formation of MuHN/MeH-eEF1A disulfide-linked multimers *in vivo*, however some differences between membrane protein fractions and insoluble fractions after treatment with non-ionic detergent (Figure 3) were observed. Centrifugation for 45 min. at 16000 × g was required to sediment MuHN/MeH and eEF1A aggregates from membrane fractions (Figure 3, fraction 3) dissolved in IEF buffer without reducing agent, whereas 10 min. at 10000 × g was sufficient in the case of last insoluble fractions (Figure 3, fraction 7) treated with IEF buffer without DTT. Moreover, IEF buffer with reducing agent solubilized virtually all protein aggregates in membrane fractions, while after fractionation insoluble multimers in fraction 7 were resistant to this treatment and could be solubilized only by using guanidine hydrochloride or SDS as denaturants. Formation of large aggregates *in vitro *during fractionation was caused by non-ionic detergent as its exclusion from washing buffers prevented enlargement of insoluble multimers. We suggest that observed *in vitro *aggregation occurs due to association between MuHN or MeH hydrophobic tails removed from membranes by non-ionic detergent. Analysis of membrane fractions by ice-cold sodium carbonate treatment [28] confirmed that recombinant MuHN and MeH are present in the form of integral membrane proteins in yeast.

These results demonstrate that viral surface proteins in yeast cytoplasm form separate aggregates, which are embedded in membranes. The core of these aggregates consists of MuHN or MeH disulfide-linked multimers involving eEF1A and is closely associated with sHsps that can be removed only under denaturing conditions. Complexes of large Hsps seem to be bound to aggregate core peripherally as they can be easily removed at high salt concentrations. *In vivo *intact aggregations of recombinant viral proteins could be characterized as "huge", because centrifugation at 16,000 × g for 5 min. was sufficient to pellet these structures from the lysates after disruption of cells in phosphate buffer.

eEF1A involvement in the core of MuHN and MeH aggregates raised a question about the regulation of eEF1A expression in observed cellular stress. As mentioned above, denaturing IEF buffer solubilized virtually all protein aggregates when applied directly to cellular membranes untreated with non-ionic detergent, thus it should be possible to separate eEF1A on 2D gels and evaluate its expression change. According to reported proteomic studies eEF1A is one of the most abundant proteins in yeast expressed at more than 500,000 copies per cell [29,30]. However, the basic nature of eEF1A (pI 9.14) is a problem in such analysis. In our study, using conventional 2D technique based on Immobilized pH gradient (IPG) strips (Invitrogen pH3-10 strips were used) this protein seemed to be underrepresented and we did not find significant eEF1A expression change in whole cell lysates (Additional file 1, Figure S1, A and B). Highly basic proteins tend to precipitate in IPG strips when their charge becomes zero, therefore in earlier proteomic studies IEF was interrupted before equilibrium to avoid eEF1A loss [29]. To overcome this problem we used Non-equilibrium pH gradient gel electrophoresis (NEPHGE) in first dimension and compared cellular protein amounts in MeH expressing and control cells. In the pI range of 4-7 it revealed similar protein expression pattern as using IPG strips, with predominant Ssa protein spots in MeH expressing *S. cerevisiae *cells, whereas in the basic pI range new strongly overexpressed protein spot corresponding to eEF1A was detected (Additional file 1, Figure S1, D). It suggests that eEF1A expression analysis using IPG strips resulted in substantial loss of this protein and was unreliable. We did not assay eEF1A expression fold change in NEPHGE based 2D experiments (see Additional file 1, Figure S1 legend), however the upregulation of eEF1A expression in response to synthesis of recombinant viral protein is evident. Moreover, according to the protein amounts, eEF1A and Ssa proteins seem to be most abundantly overexpressed during cell stress (Additional file 1, Figure S1). This data indicates that eEF1A plays an important role in the specific stress response in *S. cerevisiae *cells.

## Discussion

We report on the overexpression of human virus surface proteins in yeast. Using powerful promoters we were able to achieve a high level of expression of MuHN and MeH. However, these recombinant viral proteins are not useful for diagnostic purposes or development of vaccines since they remain totally insoluble and inactive. It is noteworthy that analogous construction of *MeH *gene was successfully overexpressed in mammalian cells at even higher level than in yeast, but producing biologically active recombinant protein [16]. Overexpression of MuHN and MeH in *S. cerevisiae*, as well as MeH in *P. pastoris*, results in the accumulation of unglycosylated protein precursors within the yeast cytoplasm. Unglycosylated MeH molecules are not detected in the preparations of this protein after synthesis in mammalian expression system [16], however the expression of the MeH protein in insect cells by baculovirus system resulted in a large amount (approximately half of the total product corresponding to 65 kDa species) of unglycosylated MeH protein molecules [31]. The recombinant MeH protein, produced in transgenic carrot plants also had lower molecular weight than the viral protein, suggesting a different glycosylation pattern, but this was not further explored [32]. It appears that maturation of MeH protein is increasingly impaired switching from mammalian cell culture to less complex expression systems, and in lower eukaryotes (e.g. yeast) we eventually observe a majority of MeH molecules in immature form of non-glycosylated precursors. However, we found that minor amounts of MeH and MuHN proteins are yet glycosylated in yeast (Figure 2D). It indicates that small amounts of viral protein precursors are successfully translocated into ER lumen and glycosylated, but this process is rather ineffective in yeast compared to higher eukaryotes.

Aggregation of unglycosylated viral protein precursors with cytoplasmic yeast proteins demonstrates that the vast majority of recombinant product is localised in the cytoplasm of yeast cells. Proteomic analysis of *S. cerevisiae *cells expressing MuHN and MeH revealed a specific stress response that, according to the list of induced proteins, is similar to recently reported cytosolic unfolded protein response (UPR-Cyto) [5]. A key feature of this response in our study is the formation of extremely large aggregates involving macromolecular structures of eEF1A, which not only interacts with viral protein precursors, but also is upregulated together with other major stress proteins. The eukaryotic translation elongation factor 1A (eEF1A, encoded by two identical yeast genes *TEF1 *and *TEF2*, also known as Tef2p) is localized in cell cytoplasm and has several important functions including delivery of aminoacyl-tRNA to the elongating ribosome [33], cytoskeleton organization via actin filament-binding and -bundling activities [34], quality surveillance of newly synthesized proteins [35] and ubiquitin-dependent degradation [36]. It was shown that mammalian homolog of yeast eEF1A protein has chaperone-like activity and, unlike other ribosome-associated factors, interacts with unfolded polypeptide chains after their release from the ribosome [35]. This data as well as reports that eEF1A promotes degradation of cotranslationally damaged proteins [36] imply that eEF1A play a role in quality surveillance of newly synthesized proteins. Our findings suggest that eEF1A may participate in both protein quality control and cellular stress response in yeast. The function of eEF1A in stress response in yeast cells has not been described before. Interestingly, eEF1A appears to be involved in heat shock response in mammalian cells where both heat shock RNA-1 (HSR1) and eEF1A are required for activation of the heat-shock transcription factor 1 (HSF1) [37]. Recently it was shown that *Legionella pneumophila *protein SidI, toxic to eukaryotic cells, specifically interacts with eEF1A in mammalian cells and this interaction induces host stress response via eEF1A-mediated activation of the HSF1 [38]. It seems likely that eEF1A interactions with MuHN and MeH precursors may result in activation of stress response in yeast utilizing a similar mechanism as in mammalian cells.

Causton *et al. *[39] have shown that environmental changes such as nutrition, temperature, pH, oxidation and osmolarity induce the expression of both sHsps and large chaperones. In contrast, in the response to synthesis of recombinant MuHN and MeH, only the large Hsps were upregulated whereas sHsps were not. The same phenomenon was observed in UPR-Cyto where similar set of large Hsps were induced and the only significantly repressed protein was a small Hsp12p [5]. The latter study has provided key evidence that the UPR-Cyto is a specific *HSF1*-mediated module of the eukaryotic heat-shock response, because most differentially regulated proteins (20 of 25 identified, including repressed sHsp) are *HSF1 *targets. The discrepancy between large Hsps and sHsps may be related to the induction of sHsp genes by transcription factors Hsf1 and Msn2/4, which play a major role in the transcriptional activation of stress-induced genes in *S. cerevisiae*. It is known that the contribution of Hsf1p and Msn2/4p is different depending on the gene and the stress condition. In fact, Hsp26 expression is induced by Hsf1p only during heat shock and depends on Msn2/4p under other stress conditions, whereas the expression of large chaperone Hsp104 depends on Hsf1p for all the conditions tested [40]. We speculate that eEF1A interactions with MuHN and MeH precursors may result in activation of Hsf1p by eEF1A via a similar mechanism as in mammalian cells [37,38], whereas Msn2/4 pathway is not activated. Then only large chaperones but not Hsp26 might be upregulated by Hsf1p, because under standard conditions recombinant proteins are expressed in non-heat-shocked yeast cells. Therefore, the involvement of eEF1A may explain the mechanism for UPR-Cyto induction in yeast. Further studies are necessary to provide evidence supporting this proposal.

In contrast to mammalian homologues, Hsp26 from *S. cerevisiae *is a temperature-regulated chaperone [41,42]. Only after exposure to elevated temperature is Hsp26 able to bind unfolded polypeptides and prevent their aggregation [43]. In our experiment Hsp26 was able to bind unfolded MuHN and MeH polypeptides at 30°C, however this did not prevent their aggregation into insoluble complexes. This data demonstrate that overexpression of human virus surface glycoproteins in yeast induces a cytoplasmic stress response that differs from heat-shock and other environmental stresses. Whereas the sHsps are upregulated under most stress conditions, here they were either not upregulated or indeed down-regulated. The usual mode of action of sHsps is to bind unfolded nascent proteins and prevent the formation of aggregates in cooperation with large Hsps, that was observed when endogeneous marker proteins are subjected to heat stress [44,45]. In contrast, recently it was demonstrated that Hsp70 and Hsp26 have opposite biological effects on the expression of mutant human cystathionine β-synthase protein in *S. cerevisiae *grown under normal temperature conditions [46]. In our case, the sHsps bound the human virus proteins-eEF1A multimers irreversibly and promoted formation of large insoluble aggregates which could not be prevented by the binding of soluble large Hsps. Thus during the synthesis of MuHN and MeHN in yeast, the usual pattern of both large and sHsp acting in concert to prevent insoluble aggregates formation and keeping unfolded proteins in a competent form for re-folding, was not observed. Irreversible process earlier was also suggested for UPR-Cyto. At late time points, heat shock and UPR-Cyto stress do differ slightly, where UPR-Cyto stress shows a unique reinduction of the chaperones, suggesting that this stress, unlike heat stress, is persistent and unrepairable [4].

We observed similar interaction between eEF1A and recombinant viral proteins as well as accumulation of major cytoplasmic stress proteins in the cases of rubella virus glycoprotein E1 and SARS coronavirus spike glycoprotein expression in *S. cerevisiae *(our unpublished data). It suggests that similar cytoplasmic stress response is not limited only to the viral surface proteins from *Paramyxoviridae *family viruses, but may also be induced by the expression of human virus surface glycoproteins of other genera in yeast. Furthermore, 2D electrophoresis experiments with various *P. pastoris *transformants expressing MeH protein showed similar pattern of overexpressed cellular proteins suggesting similar stress response as in *S. cerevisiae *(not shown). However, identification of the proteins by MS in *P. pastoris *has not been done, therefore the UPR-Cyto described in this paper refers to *S. cerevisiae*, rather than to yeast in general. As recently *P. pastoris *genome sequence has become publicly available [47,48] it will be more convenient to perform proteomic analysis including MS protein identifications in this species.

Our study is focused on the effects of overexpression of the full-length MuHN and MeH proteins including hydrophobic N-terminal signal anchor transmembrane (TM) domain, which serves as both a signal sequence and a TM-spanning sequence. Native viral surface protein precursors are transported through ER membrane cotranslationally and depend on SRP, whereas significant posttranslational insertion into ER membrane does not occur [49]. The use of either SRP-dependent cotranslational or SRP-independent posttranslational translocation pathway in yeast is determined by signal sequence hydrophobicity [50]. Hydrophobicity plots show that signal sequences of SRP-independent substrates have peaks that do not exceed +2.0 U (as defined by Kyte and Doolittle [51]), whereas those that use SRP have peaks approaching +3.0 U [50]. As viral surface proteins possess highly hydrophobic signal sequences, e.g. hydrophobicity of MeH and MuHN TM domains peaks over 3.0 U, they should use SRP-dependent translocation pathway in yeast cells. It is consistent with our results showing that the replacement of MeH native TM domain by *S. cerevisiae *α-factor signal sequence greatly enhances the amount of translocated and glycosylated viral protein (Figure 4). It is well established that prepro-α-factor is translocated posttranslationally by SRP-independent route [50], thus increased translocation rate may be simply explained by higher protein load in *S. cerevisiae *posttranslational versus SRP-dependent translocation pathway. Massive expression of the native MuHN and MeH precursors may overload the SRP-dependent translocation pathway and result in the emergence of free nascent chains in the cytosol. On the other hand, there are various accessory factors, such as TRAM (translocating chain-associating membrane protein) or TRAP (translocon-associated protein complex), that interact directly with the nascent chain to stabilize the looped orientation essential for effective cotranslational translocation in mammalian cells [for review: [52]]. For example, it was shown that the vesicular stomatitis virus G protein TM segment interacts with TRAM during cotranslational protein integration into the ER membrane [53]. Yeast have no homologues for TRAM, TRAP and some other mammalian accessory factors, therefore SRP-dependent cotranslational translocation of viral protein nascent chains into ER may be underresourced in yeast cells.

Taken together, our data suggest that MuHN and MeH synthesis is inefficient mostly due to bottleneck in translocation of viral protein precursors and induces specific cytoplasmic stress leading to accumulation of insoluble protein aggregates in yeast cells. We see two possible reasons for inefficient translocation of viral surface proteins in yeast: (1) limited capacity of SRP-dependent translocation pathway; (2) lack of mammalian accessory factors for translocation. The idea that membrane protein production exceeds the capacity of the Sec translocon and this bottleneck may be alleviated by co-expression of Sec translocon components comes from membrane protein overexpression studies in *E. coli *[54]. Recently no evidence for this was found in yeast, where *SEC63 *co-expression and *SEC102 *deletion did not give improved recombinant membrane protein yield [55]. However, that manipulation could enhance only posttranslational translocation. It should be worthwhile to co-express the components of SRP-dependent pathway, including mammalian accessory factors, such as TRAM or TRAP. Any successful manipulation increasing the translocation rate of recombinant product should lower the formation of aggregates in the cytoplasm and minimize UPR-Cyto activation upon membrane protein overexpression. Thus, cytosolic UPR appears as a marker for inefficient translocation of overexpressed viral surface glycoprotein precursors in *S. cerevisiae *cell factory. It is yet unclear whether the set of induced cellular proteins acting in UPR-Cyto stress is the same for all misfolded proteins overexpressed in cytoplasm or if there are significant differences in response to specific protein groups, e.g. human virus surface proteins. Further studies are needed to determine the exact mechanism of this cellular response in yeast.

## Conclusions

Here we show that overexpression of human virus surface protein precursors induces cytosolic unfolded protein response (UPR-Cyto) in yeast *S. cerevisiae*. Yeast cells are able to transfer the native sequence MeH and MuHN polypeptides into ER lumen, however, in contrast to mammalian cells, this process is inefficient and only a small amount of nascent chains is translocated and glycosylated. The majority of synthesized viral protein accumulates as unglycosylated precursors in the cytosol and forms insoluble aggregates with stress proteins, including Hsps and eEF1A. Yeast cell response to overexpressed heterologous membrane protein precursors in the cytosol has not been previously studied. We found that this response corresponds to recently defined UPR-Cyto, which represent a subset of proteins involved in the heat-shock response. According to our data and to another recent study [5], the UPR-Cyto may be characterized by different action and regulation of small Hsps versus large chaperones of Hsp70, Hsp90 and Hsp110 families. Furthermore, our results suggest an important role for eEF1A in this cellular response. Involvement of eEF1A may explain the mechanism by which only large chaperones, but not small Hsps are upregulated in the UPR-Cyto.

In conclusion, our study highlights important differences between viral surface protein expression in yeast and mammalian cells at the first stage of secretory pathway. Based on our observations, we consider a few reasons for inefficient translocation of viral protein precursors through ER membrane in yeast. Here we also propose possible strategies to overcome this limitation of yeast expression system.

## Methods

### Viral genes and plasmids

Apart from the primers used, the cloning strategy of mumps virus *HN *(*MuHN*) and measles virus *H *(*MeH*) genes was essentially identical to that used for cloning mumps virus *NP *and measles virus *N *genes, respectively [17,18]. To facilitate detection and purification of recombinant product the histidine-tag (his-tag) sequence was added in-frame into the 3' end of the *MeH *gene by PCR using the oligonucleotide primer encoding hexamerous histidines (his6). It was earlier shown that the his-tagged recombinant MeH protein, expressed in mammalian cells, bears all the biological activities of the wild-type protein [16]. No published data was available about the his-tagged MuHN protein, therefore we expressed both the his-tagged and native sequence *MuHN *gene variants. All DNA manipulations were performed according to standard procedures [56]. Enzymes and kits for DNA manipulations were purchased from Fermentas UAB (Vilnius, Lithuania). The measles virus *H *(*MeH*) gene was amplified by PCR from cDNA after extraction from and reverse transcription of reconstituted Priorix vaccine, containing the measles Schwarz strain (GlaxoSmithKline, UK), (GenBank AF266291). The mumps virus *HN *(*MuHN*) gene was amplified by RT-PCR from the wild-type mumps virus Gloucester strain isolated in the UK (GenBank AF280799). Primers used in amplification of *MeH *included a *Spe*I and those used in the amplification of *MuHN *- a *Xba*I site for subcloning into the yeast vectors, a single ATG codon in the forward primer and a stop TAA codon in the reverse primer. The following primers containing the *Spe*I or *Xba*I sites (in bold) and the start and stop codons (underlined) were used.

Primers for the *MeH *gene:

Forward (5'→3') aat **act agt **aca atg tca cca caa cga gac

Reverse (5'→3') aat **act agt **tta atg gtg atg gtg atg gtg tct gcg att ggt tcc atc

Primers for the *MuHN *gene:

Forward (5'→3') aca **tct aga **ata atg gag ccc tcg aaa ttc

Reverse (5'→3') atc ggg ccc **tct aga **tta agt gat agt caa tct agt tag (for cloning of the native *MuHN *sequence)

Reverse (5'→3') atc **tct aga **tta atg gtg atg gtg atg gtg agt gat agt caa tct agt tag (for cloning of *MuHN *with His6-tag at the C-terminus)

Bands corresponding to the *MeH *and *MuHN *genes were gel-purified and cloned into pCR^® ^2.1 TOPO^® ^(Invitrogen), sequenced and used for cloning into the yeast *S. cerevisiae *vectors under the control of galactose inducible promoters or into the *P. pastoris *vectors under the control of methanol inducible *AOX1 *promoter. The *MeH *gene was cloned into the *Spe*I site of the *S. cerevisiae *vector pFGG3 [18] and the *MuHN *gene - into the *Xba*I site of the vector pFX7 [17], respectively. The resulting plasmids pFGG3-MeH, pFX7-MuHN and pFX7-MuHN-His6 were used for the transformation of the *S. cerevisiae *strains as described previously [17]. Both *MuHN *and *MeH *genes were also cloned into the *Avr*II site of *P. pastoris *vector pPIC3.5K (Invitrogen) for intracellular expression under the control of methanol inducible *AOX1 *promoter. For the secreted expression the 5' parts of viral genes encoding N-terminal TM anchor domains were removed by restriction digest with *Bpi*I in *MuHN *(codon 67) and *Xba*I in *MeH *(codon 78), and the rest of the genes were in frame fused with 269 bp fragment encoding the α-factor signal sequence by cloning into *P. pastoris *vector pPIC9K. The fusion sites were verified by sequencing. The resulting plasmids pPIC3.5K-MuHN and pPIC9K-MuHN were linearized with *Bgl*II, whereas pPIC3.5K-MeH and pPIC9K-MeH with *Dra*I, and used for electroporation into *P. pastoris *GS115 [57].

### Yeast strains, media and growth

*S. cerevisiae *strain AH22 (*MATa leu2-3 leu2-112 his4-519 can1 *[*KIL-o*]) was used for expression of cloned *MuHN *and *MeH *genes under the control of galactose inducible promoters. Some expression experiments were also carried out in protease-deficient AH22 derivative ∆*pep4 *strain and wild-type *S. cerevisiae *strain FH4 (*MATa/α*). Similar results were achieved in all strains tested, but additional data was not included into calculations. All quantitave data presented in this report was obtained from AH22 strain only.

*S. cerevisiae *cells were grown in YEPD medium (yeast extract 1%, peptone 2%, and glucose 2%) supplemented with 5 mM formaldehyde or in induction medium YEPG (yeast extract 1%, peptone 2%, and galactose 3%). The procedure used for expression of viral surface proteins was similar to that for mumps and measles nucleocapsid proteins described earlier [17,18]. Briefly, *S. cerevisiae *cells harbouring plasmids with viral genes were inoculated into YEPD media supplemented with 5 mmol formaldehyde, grown overnight, re-inoculated into YEPG induction media and cultured at 30°C for 16 h. Cells were harvested by centrifugation and stored at -70°C. Wet biomass accumulation was calculated as the ratio between the weights of pelleted cells after and before induction in galactose medium. The growth rate in liquid cultures was also monitored by A_600 _measurements. Doubling times were determined during the period of exponential growth in YEPG media.

The strain GS115 *his4 *(Invitrogen, Groningen, The Netherlands) was used for the expression of viral genes in *P. pastoris*. Transformation of *P. pastoris *GS115 *his4 *was performed by electroporation (Bio Rad, Gene Pulser) according to Cregg and Russell [57]. *P. pastoris *His^+ ^transformants were selected on a minimal agar medium (0.67% YNB, 2% glucose). Transformants with a high copy number were selected on YEPD-agar plates containing 0.5-1.5 mg/ml G418 (Amresco, Solon, USA). The media and growth conditions for selected *P. pastoris *transformants were used essentially as suggested by the manufacturers, the detailed protocols were described earlier [18,58].

### Preparation of yeast lysates, SDS-PAGE and Western blotting

10-20 mg of cell pellets were collected into a 1.5 ml microcentrifuge tube by centrifugation, washed with distilled water and resuspended in 10 volumes (vol/wt) of breaking buffer containing 50 mM sodium phosphate, pH7.2, 5 mM EDTA and 1 mM PMSF. An equal volume of glass beads was added and the cells were lysed by vortexing at high speed, 8 times for 30 sec, with cooling on ice for 30 sec between each vortexing. Then an equal volume (to that of breaking buffer) of 2×SDS-PAGE sample buffer (125 mM Tris-HCl, pH6.8, 20% glycerol, 8% SDS, 150 mM DTT, 0.01% bromophenol blue) was added directly to the same tube, mixed and boiled immediately at 100°C for 10 minutes. 4 μl of the prepared whole cell lysate was loaded onto SDS-polyacrylamide gel (up to 20 μg protein in each lane) and gel electrophoresis was run in SDS-Tris-glycine buffer. Samples of the fractions obtained during fractionation of yeast lysates were diluted to protein concentration <10 μg/μl, mixed with an equal volume of 2× SDS-PAGE sample buffer, boiled and subjected to SDS-PAGE and Western blotting. Protein concentrations were determined by Roti-Nanoquant Protein-assay (Carl Roth Gmbh.), which is a modification of Bradford's protein assay.

The proteins in PAA gels were stained by adding Coomassie brilliant blue R-250. After SDS-PAGE, the proteins were transferred to the nitrocellulose membrane Hybond™; ECL (Amersham, UK) as described in [56] and incubated with antibodies according to the manufacturers' recommendations. Anti-His antibodies (mouse monoclonal Tetra-His Antibody) were purchased from QIAGEN (USA). Ascite fluids containing monoclonal antibodies against MuHN were kindly provided by Dr. Claes Örvell (Karolinska Universitetssjukhuset, Huddinge, Sweden). Horseradish peroxidase (HRP)-labelled goat anti-mouse IgG conjugates (Bio-Rad) were used for the detection of specific antibody-binding. HRP-labelled lectin from Concanavalin A (*Canavalia ensiformis*) was purchased from Sigma and used for probing sugar side chains on immobilized proteins as recommended by the manufacturers. Digestion with PNGase F was performed using a kit from New England BioLabs.

Quantitative evaluation of protein bands in 1D blots and gels was performed using the ImageQuant TL 1D gel analysis software (GE Healthcare).

### Fractionation of yeast lysates

A step-wise diagram for fractionation of yeast lysates is shown in Figure 3. Crude cell extracts were prepared using glass beads (Sigma, 0.5 mm) in 2 volumes of breaking buffer. Cells were lysed by vortexing at high speed, 16 times for 30 sec with cooling on ice for 30 sec between each vortexing. Crude lysates were precleared by centrifuging them at 800 × g for 5 min at 4°C to remove unlysed cells. Clarified lysates (fraction 1) were further fractionated by centrifugation at 16000 × g for 45 min at 4°C. After the first centrifugation the supernatant ("totally soluble" fraction 2) was retained, the pellets ("membrane protein" fraction 3) were resuspended in breaking buffer with 1% Triton X-100 for 1 h on ice and centrifuged again. The supernatant containing fraction 4 of proteins soluble in non-ionic detergent was collected, whereas insoluble pellets (fraction 5) were resuspended in breaking buffer containing 1M NaCl and 1% Triton X-100, pH8.0 for 1 h on ice. Then proteins soluble at high salt concentration (fraction 6) were separated from the insoluble protein fraction by centrifugation as before. The remaining pellets (insoluble under native conditions, fraction 7) were resuspended in denaturing buffer B (containing 8M urea, 100 mM sodium phosphate, pH8.0, 10 mM Tris-HCl, 5% glycerol, 1% Triton X-100 and 15 mM 2-mercaptoethanol) or IEF buffer (7 M urea, 2 M thiourea, 2% CHAPS detergent, 0,5% ampholytes, 0,002% Bromphenol Blue; 75 mM DTT) for 1 h at room temperature and centrifuged at 16000 × g for 10 min at 20°C. The supernatant with solubilized proteins was analysed as fraction 8 (described as "insoluble under native conditions" in Table 1 or "soluble in IEF buffer" in Figure 3), whereas pellet fraction 9 (described as "insoluble under denaturing conditions" or "insoluble in IEF buffer" in Table 1 and Figure 3, respectively) contained partially purified recombinant viral proteins. The pellet (fraction 9) was additionally treated with various denaturing solutions (including 6M guanidine hydrochloride, 4% SDS or urea/thiourea as denaturants) and with increasing concentrations of reducing agents (2-mercaptoethanol or DTT), with subsequent centrifugation (results are given in manuscript text). Additionally membrane protein fraction 3 was directly resuspended in denaturing IEF buffer with or without reducing agent (DTT) and examined as described in Results section. All fractions obtained during fractionation of yeast lysates were analysed by SDS-PAGE and Western blotting. Soluble protein fractions were also used for two-dimensional electrophoresis.

### Two-dimensional (2D) gel electrophoresis

For comparative analysis all experiments with MuHN or MeH expressing and control cells were run in parallel. Cells were lysed by mechanical disruption using glass beads in denaturing IEF buffer (7 M urea, 2 M thiourea, 2% CHAPS detergent, 0,5% ampholytes, 0,002% Bromphenol Blue; 75 mM DTT), cell debris were removed by centrifugation at 16000 × g for 15 min. at 16°C. Supernatants (whole cell lysates) were applied onto 7 cm length IPG strips. Invitrogen ZOOM IPGRunner system was used for IEF according to manufacturer's recommendations. Soluble fractions, achieved during fractionation of yeast lysates under native conditions, were added to IEF buffer directly or (in the case of high salt concentration) treated with 2-D Clean-Up Kit (Amersham Biosciences) prior to use. After IEF the strips were incubated in equilibration buffer (50 mM Tris-HCl pH 8.8, 2% SDS, 6 M urea, 30% glycerol, 0,002% Bromphenol Blue) containing, in course, reducing (75 mM DTT) and alkylating (125 mM 2-iodoacetamyde) agents (treated for 15 min. by both). Equilibrated strips were applied onto SDS-polyacrylamide gels and SDS-PAGE was run for the second dimension. Proteins in 2D gels were visualised with Coomassie brilliant blue R-250. Additional non-equilibrium pH gradient gel electrophoresis (NEPHGE) experiments were performed using WITA VISION i2D System as recommended by manufacturers.

### Analysis of 2D gel images

2D gel images were analysed using the ImageMaster 2D Platinum 7.0 software (GE Healthcare). Detected protein spots were quantified by the relative volumes (%Vol), indicating percentage of volumes of a separate spots among volume of all protein spots in a gel. Protein expression pattern and differences between MuHN/MeH expressing and control cells were examined using factor analysis in the same software. Protein spots were selected for identification from those matches, which were most important in determining difference between MuHN/MeH and control axes in Factor Projection Report given by the software. MuHN/MeH influence on the expression level of identified proteins was evaluated by calculating "fold change" - the ratio of %Vol between spots of MuHN/MeH expressing and control cells, respectively. Fold changes given in Table 1 represent data from three independent experiments in *S. cerevisiae *strain AH22. All identified proteins showed similar expression fold changes upon either MuHN or MeH overexpression, therefore a single mean ± standard deviation (SD; n = 6) was calculated from six values of fold changes (three MuHN/control and three MeH/control) for each spot.

### Tryptic In-Gel Digestion

Protein identification was carried out by the company WITA (Teltow, Germany) by means of tryptic digestion and MALDI-PMF or nanoLC-ESI-MS/MS. For tryptic in-gel digestion the spots were cut and reduced with 100 mM DTT, dehydrated at 50% (v/v) and 80% (v/v) acetonitrile, treated overnight with 50 ng of MS grade trypsin (Roche Diagnostics) in a buffer prepared from 25 mM ammonium bicarbonate (pH 8) at 37°C. Peptide extraction was performed with 20 μl 0.2% (v/v) tri-fluoro-acetic acid, 20% (v/v) acetonitrile and 50% (v/v) acetonitrile. The peptide mixture was lyophilized and re-dissolved in 0.2% (v/v) tri-fluoro-acetic acid.

### MALDI-PMF

The samples were desalted by C18-ZipTips (Millipore), spotted onto a 400/384 AnchorChip-Target (Bruker Daltonics) and mixed with 0.8 μl of matrix solution (HCCA, α-Cyano-4-hydroxycinnamic acid) on target. The spots were dried thoroughly and re-crystallized with methanol.

Data collection was performed automatically on Bruker Reflex III MALDI mass spectrometer. The range of measurement was set to 0 - 4 kDa. Main instrument parameters were: reflector positive mode, 25 kV and matrix deflection 0 - 600 Da.

Analysis and post-processing of the spectra was performed employing XMASS/NT 5.1.5 and Flex Analysis 2.0 software (Bruker Daltonics). Protein identification was performed via Bio-Tools 2.2 (Bruker Daltonics) and Mascot Server 2.0 (Matrix Science) in peptide mass fingerprint search mode using the MSDB as well as the NCBInr database.

### nLC-ESI-MS/MS

The total volume of the samples was injected into the nLC-ESI system. Data collection was performed automatically on a Bruker Esquire HCT mass spectrometer using the Hystar 2.3 and EsquireControl software. Analysis and post-processing of spectra was performed employing HyStar PP 2.3 and DataAnalysis 5.4 (Bruker Daltonics). Protein identification was performed via Bio-Tools 2.2 (Bruker Daltonics) and Mascot Server 2.0 (Matrix Science) in MS/MS ion search mode using the NCBInr database.

## Competing interests

The authors declare that they have no competing interests.

## Authors' contributions

EČ was involved in all aspects of the experimental design, data collection, analysis and interpretation. DS has cloned viral genes, carried out the immunoassays and edited the manuscript. MJ participated in expression work, coordinated the purification experiments and analysed the data. KS initiated the study, participated in its design and coordination and helped to draft the manuscript. RS conceived of the study, was involved in all aspects of the experimental design, data collection, analysis and interpretation, and drafted the manuscript. All authors contributed to the final version of the manuscript.

## Supplementary Material

Additional file 1 **Supplemental Data**. Protein identification data and Supplemental Figure S1. Click here for file
