# Supplementary material for: Overexpression of human virus surface glycoprotein precursors induces cytosolic unfolded protein response in Saccharomyces cerevisiae
Source: Microb Cell Fact. 2011 May 19;10:37. doi: 10.1186/1475-2859-10-37 (PMC3120639; doi:10.1186/1475-2859-10-37)
Supplement: Additional file 1 — Supplemental Data. Protein identification data and Supplemental Figure S1. [file 1475-2859-10-37-S1.PDF]

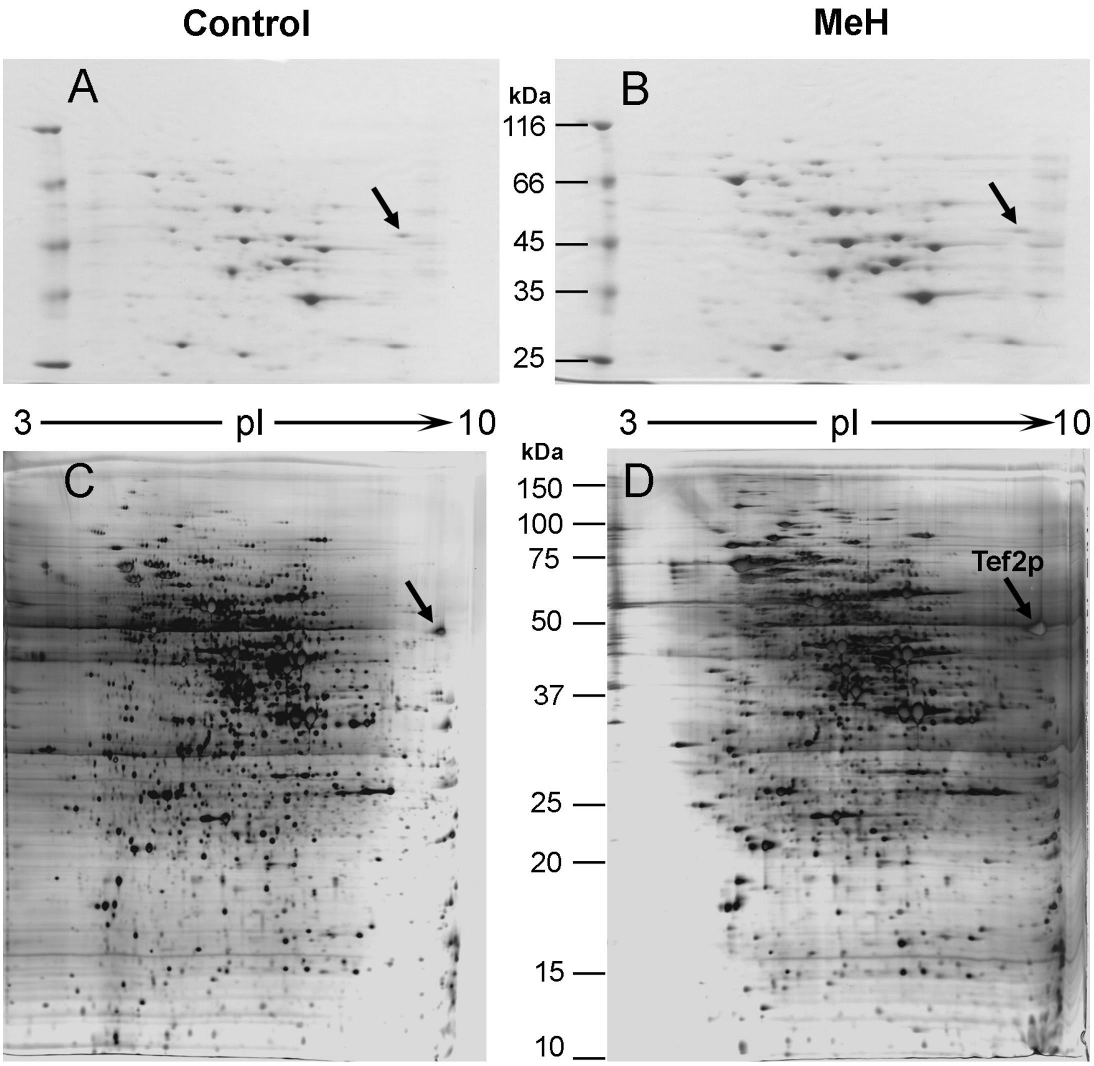

Supplemental Figure S1. Whole cell lysates were separated by 2DE, based on IPG strips (A, B) and NEPHGE (C,D). The same samples from MeH expressing (B, D) and control cells (A, C) were analysed by Invitrogen ZOOM IPGRunner system (A, B; 7 cm length IPG strips, pH 3-10; 2D gels were Coomassie stained) or by WITA VISION i2D system (C, D; 22,5 cm length NEPHGE gels, pH 3-10; 2D gels were silver stained), as recommended by the manufacturers. The main difference between results obtained using these systems is the overexpression of the highly basic eEF1A (identified as Tef2p) protein shown by NEPHGE based method, but not shown by using IPG strips (spots corresponding to eEF1A are marked in all gels with arrow). We were unable to determine the exact eEF1A expression fold change in the NEPHGE based experiment due to overexpression of eEF1A, resulting in an oversaturated eEF1A spot in silver stained gels (D). Qualitative analysis of eEF1A expression will be reported elsewhere.

Protein identification

Spot 1 (MALDI fingerprint)

Proteins identified: **Ssa1p** and **Ssa2p**

Ssa1

```
Mowse Score: 76 (threshold 72)
Nominal mass (Mr): 69484; Calculated pI value: 5.00
Number of mass values matched: 10
Sequence Coverage: 19%

Matched peptides shown in Bold Red

      1 SKAVGIDLGT TYSCVAHFAN DRVDIIANDQ GNRTTPSFVA FTDTERLIGD
     51 AAKNQAAMNP SNTVFDAKRL IGRNFNDPEV QADMKHFPFK LIDVDGKPQI
    101 QVEFKGETKN FTPEQISSMV LGKMKETAES YLGAKVNDAV VTVPAYFNDS
    151 QRQATKDAGT IAGLNVLRII NEPTAAAIAY GLDKKGKEEH VLIFDLGGGT
    201 FDVSLLSIED GIFEVKATAG DTHLGGEDFD NRLVNHFIQE FKRKNKKDLS
    251 TNQRALRRLR TACERAKRTL SSSAQTSVEI DSLFEGIDFY TSITRARFEE
    301 LCADLFRSTL DPVEKVLRDA KLDKSQVDEI VLVGGSTRIP KVQKLVTDYF
    351 NGKEPNRSIN PDEAVAYGAA VQAAILTGDE SSKTQDLLLL DVAPLSLGIE
    401 TAGGVMTKLI PRNSTIPTKK SEIFSTYADN QPGVLIQVFE GERAKTKDNN
    451 LLGKFELSGI PPAPRGVPQI EVTFDVDSNG ILNVSAVEKG TGKSNKITIT
    501 NDKGRLSKED IEKMVAEAEK FKEEDEKESQ RIASKNQLES IAYSLKNTIS
    551 EAGDKLEQAD KDTVTKKAEE TISWLDSNTT ASKEEFDDKL KELQDIANPI
    601 MSKLYQAGGA PGGAAGGAPG GFPGGAPPAP EAEGPTVEEV D
```

Ssa2

```
Mowse Score: 76 (threshold 72)
Nominal mass (Mr): 69296; Calculated pI value: 4.95
Number of mass values matched: 10
Sequence Coverage: 19%

Matched peptides shown in Bold Red

      1 SKAVGIDLGT TYSCVAHFSN DRVDIIANDQ GNRTTPSFVG FTDTERLIGD
     51 AAKNQAAMNP ANTVFDAKRL IGRNFNDPEV QGDMKHFPFK LIDVDGKPQI
    101 QVEFKGETKN FTPEQISSMV LGKMKETAES YLGAKVNDAV VTVPAYFNDS
    151 QRQATKDAGT IAGLNVLRII NEPTAAAIAY GLDKKGKEEH VLIFDLGGGT
    201 FDVSLLSIED GIFEVKATAG DTHLGGEDFD NRLVNHFIQE FKRKNKKDLS
    251 TNQRALRRLR TACERAKRTL SSSAQTSVEI DSLFEGIDFY TSITRARFEE
    301 LCADLFRSTL DPVEKVLRDA KLDKSQVDEI VLVGGSTRIP KVQKLVTDYF
    351 NGKEPNRSIN PDEAVAYGAA VQAAILTGDE SSKTQDLLLL DVAPLSLGIE
    401 TAGGVMTKLI PRNSTIPTKK SEVFSTYADN QPGVLIQVFE GERAKTKDNN
    451 LLGKFELSGI PPAPRGVPQI EVTFDVDSNG ILNVSAVEKG TGKSNKITIT
    501 NDKGRLSKED IEKMVAEAEK FKEEDEKESQ RIASKNQLES IAYSLKNTIS
    551 EAGDKLEQAD KDAVTKKAEE TIAWLDSNTT ATKEEFDDQL KELQEVANPI
    601 MSKLYQAGGA PEGAAPGGFP GGAPPAPEAE GPTVEEVD
```

Spot 1 (nLC-ESI-MS/MS)

Proteins identified: **Ssa2p and Ssa1p**

Ssa2

Mowse Score: **688** (ions scores threshold 52)
Nominal mass (M_r): **69296**; Calculated pI value: **4.95**
Peptides matched: **21**
Sequence Coverage: **41%**

Matched peptides shown in **Bold Red**

1 SKAVGIDLGT TYSCVAHFSN DRVDIIANDQ GNR**TTPSFVG FTDTERLIGD**
51 **AAKNQAAMNP ANTVFDAK**RL IGR**NFNDPEV QGDMK**HFPFK LIDVDGKPQI
101 QVEFKGETK**N FTPEQISSMV LGK**MKETAES YLGAK**VNDAV VTVPAYFNDS**
151 **QR**QATKDAGT IAGLNVLRII NEPTAAAIAY GLDKKGKEEH VLIFDLGGGT
201 FDVSLLSIED GIFEVKATAG DTHLGGEDFD NR**LVNHFIQE FKR**KNKKDLS
251 TNQRALRRLR TACERAKRTL SSSAQTSVEI DSLFEGIDFY TSITRARFEE
301 LCADLFR**STL DPVEK**VLRDA KLDK**SQVDEI VLVGGSTR**IP KVQK**LVTDYF**
351 **NGKEPNRSIN PDEAVAYGAA VQAAILTGDE SSK**TQDLLLL DVAPLSLGIE
401 TAGGVMTK**LI PR**NSTIPTKK SEVFSTYADN QPGVLIQVFE GERAK**TKDNN**
451 **LLGKFELSGI PPAPR**GVPQI EVTFDVDSNG ILNVSAVEKG TGKSNK**ITIT**
501 **NDKGR**LSKED IEKMVAEAEK FKEEDEKESQ R**IASKNQLES IAYSLKNTIS**
551 **EAGDKLEQAD KDAVTK**KAEE TIAWLDSNTT ATKEEFDDQL K**ELQEVANPI**
601 **MSKLYQAGGA PEGAAPGGFP GGAPPAPEAE GPTVEEVD**

Ssa1

Mowse Score: **618** (ions scores threshold 52)
Nominal mass (M_r): **69725**; Calculated pI value: **5.00**
Peptides matched: **18**
Sequence Coverage: **30%**

Matched peptides shown in **Bold Red**

1 MSKAVGIDLG TTYSCVAHFA NDRVDIIAND QGNR**TTPSFV AFTDTERLIG**
51 **DAAKNQAAMN PSNTVFDAK**R LIGRNFNDPE VQADMKHFPF KLIDVDGKPQ
101 IQVEFKGETK **NFTPEQISSM VLGK**MKETAE SYLGAK**VNDA VVTVPAYFND**
151 **SQR**QATKDAG TIAGLNVLRI INEPTAAAIA YGLDKKGKEE HVLIFDLGGG
201 TFDVSLLFIE DGIFEVKATA GDTHLGGEDF DNR**LVNHFIQ EFKR**KNKKDL
251 STNQRALRRL RTACERAKRT LSSSAQTSVE IDSLFEGIDF YTSITRARFE
301 ELCADLFR**ST LDPVEK**VLRD AKLDK**SQVDE IVLVGGSTR**I PKVQK**LVTDY**
351 **FNGKEPNRSI NPDEAVAYGA AVQAAILTGD ESSK**TQDLLL LDVAPLSLGI
401 ETAGGVMTK**L IPR**NSTISTK KFEIFSTYAD NQPGVLIQVF EGERAK**TKDN**
451 **NLLGKFELSG IPPAPR**GVPQ IEVTFDVDSN GILNVSAVEK GTGKSNK**ITI**
501 **TNDKGR**LSKE DIEKMVAEAE KFKEEDEKES QR**IASKNQLE SIAYSLK**NTI
551 SEAGDKLEQA DKDTVTKKAE ETISWLDSNT TASKEEFDDK LK**ELQDIANP**
601 **IMSK**LYQAGG APGGAAGGAP GGFPGGAPPA PEAEGPTVEE VD

Spot 2 (MALDI fingerprint)

Protein identified: **Ssa1p**

Ssa1

Mowse Score: **75** (threshold 72)
Nominal mass (M_r): **69484**; Calculated pI value: **5.00**
Number of mass values matched: **10**
Sequence Coverage: **17%**

Matched peptides shown in **Bold Red**

1 SKAVGIDLGT TYSCVAHFAN DRVDIIANDQ GNR**TTPSFVA FTDTER**LIGD
51 AAKNQAAMNP SNTVFDAKRL IGRNFNDPEV QADMKHFPFK LIDVDGKPQI
101 QVEFKGETKN FTPEQISSMV LGKMKETAES YLGAK**VNDAV VTVPAYFNDS**
151 **QR**QATK**DAGT IAGLNVLR**II NEPTAAAIAY GLDKKGKEEH VLIFDLGGGT
201 FDVSLLSIED GIFEVKATAG DTHLGGEDFD NR**LVNHFIQE FKR**KNKKDLS
251 TNQRALRRLR TACERAKRTL SSSAQTSVEI DSLFEGIDFY TSITRARFEE
301 LCADLFRSTL DPVEKVLRDA KLDKSQVDEI VLVGGSTRIP KVQK**LVTDYF**
351 **NGKEPNR**SIN PDEAVAYGAA VQAAILTGDE SSKTQDLLLL DVAPLSLGIE
401 TAGGVMTKLI PRNSTIPTK**K SEIFSTYADN QPGVLIQVFE GER**AK**TKDNN**
451 **LLGKFELSGI PPAPR**GVPQI EVTFDVDSNG ILNVSAVEKG TGKSNKITIT
501 NDKGRLSKED IEKMVAEAEK FKEEDEKESQ RIASKNQLES IAYSLKNTIS
551 EAGDKLEQAD KDTVTKKAEE TISWLDSNTT ASKEEFDDKL KELQDIANPI
601 MSKLYQAGGA PGGAAGGAPG GFPGGAPPAP EAEGPTVEEV D

Spot 2 (nLC-ESI-MS/MS)

Proteins identified: **Ssa2p and Ssa1p**

Ssa2

Mowse Score: **303** (ions scores threshold 52)
Nominal mass (M_r): **69296**; Calculated pI value: **4.95**
Peptides matched: **5**
Sequence Coverage: **8%**

Matched peptides shown in **Bold Red**

1 SKAVGIDLGT TYSCVAHFSN DRVDIIANDQ GNRTTPSFVG FTDTERLIGD
51 AAKNQAAMNP ANTVFDAKRL IGR**NFNDPEV QGDMK**HFPFK LIDVDGKPQI
101 QVEFKGETKN FTPEQISSMV LGKMK**ETAES YLGAK**VNDAV VTVPAYFNDS
151 QRQATKDAGT IAGLNVLRII NEPTAAAIAY GLDKKGKEEH VLIFDLGGGT
201 FDVSLLSIED GIFEVKATAG DTHLGGEDFD NRLVNHFIQE FKRKNKKDLS
251 TNQRALRRLR TACERAKRTL SSSAQTSVEI DSLFEGIDFY TSITRARFEE
301 LCADLFRSTL DPVEKVLRDA KLDKSQVDEI VLVGGSTRIP KVQKLVTDYF
351 NGKEPNRSIN PDEAVAYGAA VQAAILTGDE SSKTQDLLLL DVAPLSLGIE
401 TAGGVMTKLI PRNSTIPTKK SEVFSTYADN QPGVLIQVFE GERAKTKDNN
451 LLGKFELSGI PPAPRGVPQI EVTFDVDSNG ILNVSAVEKG TGKSNK**ITIT**

501 **NDKGRLSKED IEK**MVAEAEK FKEEDEKESQ RIASKNQLES IAYSLK**NTIS**
551 **EAGDKLEQAD K**DAVTKKAEE TIAWLDSNTT ATKEEFDDQL KELQEVANPI
601 MSKLYQAGGA PEGAAPGGFP GGAPPAPEAE GPTVEEVD

Ssa1

Mowse Score: **290** (ions scores threshold 52)
Nominal mass (M_r): **69725**; Calculated pI value: **5.00**
Peptides matched: **6**
Sequence Coverage: **9%**

Matched peptides shown in **Bold Red**

1 MSKAVGIDLG TTYSCVAHFA NDRVDIIAND QGNRTTPSFV AFTDTERLIG
51 DAAK**NQAAMN PSNTVFDAKR** LIGRNFNDPE VQADMKHFPF KLIDVDGKPQ
101 IQVEFKGETK NFTPEQISSM VLGKMK**ETAE SYLGAK**VNDA VVTVPAYFND
151 SQRQATKDAG TIAGLNVLRI INEPTAAAIA YGLDKKGKEE HVLIFDLGGG
201 TFDVSLLFIE DGIFEVKATA GDTHLGGEDF DNRLVNHFIQ EFKRKNKKDL
251 STNQRALRRL RTACERAKRT LSSSAQTSVE IDSLFEGIDF YTSITRARFE
301 ELCADLFRST LDPVEKVLRD AKLDKSQVDE IVLVGGSTRI PKVQKLVTDY
351 FNGKEPNRSI NPDEAVAYGA AVQAAILTGD ESSKTQDLLL LDVAPLSLGI
401 ETAGGVMTKL IPRNSTISTK KFEIFSTYAD NQPGVLIQVF EGERAKTKDN
451 NLLGKFELSG IPPAPRGVPQ IEVTFDVDSN GILNVSAVEK GTGKSNK**ITI**
501 **TNDKGRLSKE DIEK**MVAEAE KFKEEDEKES QRIASKNQLE SIAYSLK**NTI**
551 **SEAGDKLEQA DK**DTVTKKAE ETISWLDSNT TASKEEFDDK LKELQDIANP
601 IMSKLYQAGG APGGAAGGAP GGFPGGAPPA PEAEGPTVEE VD

Spot 3 (nLC-ESI-MS/MS)

Protein identified: **Ssa4p**

Ssa4

Mowse Score: **478** (ions scores threshold 52)
Nominal mass (M_r): **69478**; Calculated pI value: **5.03**
Peptides matched: **13**
Sequence Coverage: **24%**

Matched peptides shown in **Bold Red**

1 SKAVGIDLGT TYSCVAHFAN DRVEIIANDQ GNRTTPSYVA FTDTERLIGD
51 AAK**NQAAMNP HNTVFDAKR**L IGR**KFDDPEV TNDAK**HYPFK VIDK**GGKPVV**
101 **QVEYK**GETKT FTPEEISSMI LTK**MKETAEN FLGTEVK**DAV VTVPAYFNDS
151 QRQATKDAGT IAGLNVLR**II NEPTAAAIAY GLDKK**SQKEH NVLIFDLGGG
201 TFDVSLLSID EGVFEVKATA GDTHLGGEDF DSRLVNFLAE EFKRKNKKDL
251 TTNQRSLRRL RTAAERAKRT LSSSAQTSIE IDSLFEGIDF YTSITRARFE
301 ELCADLFR**ST LEPVEK**VLAD SKLDKSQIDE IVLVGGSTRI PKVQKLVSDF
351 FNGKEPNRSI NPDEAVAYGA AVQAAILTGD QSSTTQDLLL LDVAPLSLGI
401 ETAGGIMTKL IPRNSTIPTK KSEVFSTYAD NQPGVLIQVF EGERTR**TKDN**
451 **NLLGKFELSG IPPAPR**GVPQ IEVTFDIDAN GILNVSAVEK GTGKSNKITI
501 TNDKGRLSKE DIDKMVAEAE KFKAEDEQEA QRVQAKNQLE SYAFTLK**NSV**

551 **SENNFK**EKVG EEDARKLEAA AQDAINWLDA SQAASTEEYK ER**QKELEGVA**
601 **NPIMSKFYGA AGGAPGAGPV PGAGAGPTGA PDNGPTVEEV D**

Spot 4 (MALDI fingerprint)

Protein identified: **Kar2p**

Kar2

Mowse Score: **106** (threshold 53)
Nominal mass (M_r): **74468**; Calculated pI value: **4.79**
Number of mass values matched: **9**
Sequence Coverage: **16%**

Matched peptides shown in **Bold Red**

1 MFFNRLSAGK LLVPLSVVLY ALFVVILPLQ NSFHSSNVLV RGADDVENYG
51 TVIGIDLGTT YSCVAVMKNG K**TEILANEQG NRITPSYVAF TDDER**LIGDA
101 AKNQVAANPQ NTIFDIKRLI GLKYNDRSVQ KDIK**HLPFNV VNK**DGKPAVE
151 VSVKGEKKVF TPEEISGMIL GKMKQIAEDY LGTK**VTHAVV TVPAYFNDAQ**
201 **R**QATK**DAGTI AGLNVLR**IVN EPTAAAIAYG LDKSDKEHQI IVYDLGGGTF
251 DVSLLSIENG VFEVQATSGD THLGGEDFDY KIVRQLIKAF KKKHGIDVSD
301 NNKALAKLKR EAEKAKRALS SQMSTRIEID SFVDGIDLSE TLTRAKFEEL
351 NLDLFKKTLK PVEKVLQDSG LEKK**DVDDIV LVGGSTR**IPK VQQLLESYFD
401 GKKASKGINP DEAVAYGAAV QAGVLSGEEG VEDIVLLDVN ALTLGIETTG
451 GVMTPLIKRN TAIPTK**KSQI FSTAVDNQPT VMIK**VYEGER AMSKDNNLLG
501 K**FELTGIPPA PR**GVPQIEVT FALDANGILK VSATDKGTGK SESITITNDK
551 GR**LTQEEIDR** MVEEAEKFAS EDASIKAKVE SRNKLENYAH SLKNQVNGDL
601 GEKLEEEDKE TLLDAANDVL EWLDDNFETA IAEDFDEKFE SLSKVAYPIT
651 SKLYGGADGS GAADYDDEDE DDDGDYFEHD EL

Spot 5 (nLC-ESI-MS/MS)

Protein identified: **Sse1p**

Sse1

Mowse Score: **810** (ions scores threshold 52)
Nominal mass (M_r): **77187**; Calculated pI value: **5.12**
Peptides matched: **17**
Sequence Coverage: **30%**

Matched peptides shown in **Bold Red**

1 STPFGLDLGN NNSVLAVARN R**GIDIVVNEV SNRSTPSVVG FGPK**NRYLGE
51 TGKNKQTSNI KNTVANLKR**I IGLDYHHPDF EQESK**HFTSK **LVELDDK**KTG
101 AEVRFAGEKH VFSATQLAAM FIDKVKDTVK QDTKANITDV CIAVPPWYTE
151 EQRYNIADAA R**IAGLNPVRI VNDVTAAGVS YGIFKTDLPE GEEKPR**IVAF
201 VDIGHSSYTC SIMAFKKGQL KVLGTACDKH FGGRDFDLAI TEHFADEFKT
251 KYKIDIRENP KAYNRILTAA EKLKKVLSAN TNAPFSVESV MNDVDVSSQL
301 SR**EELEELVK PLLER**VTEPV TKALAQAKLS AEEVDFVEII GGTTRIPTLK

```
351 QSISEAFGKP LSTTLNQDEA IAKGAAFICA IHSPTLRVRP FKFEDIHPYS
401 VSYSWDKQVE DEDHMEVFPA GSSFPSTKLI TLNRTGDFSM AASYTDITQL
451 PPNTPEQIAN WEITGVQLPE GQDSVPVKLK LRCDPSGLHT IEEAYTIEDI
501 EVEEPIPLPE DAPEDAEQEF KKVTKTVKKD DLTIVAHTFG LDAKKLNELI
551 EKENEMLAQD KLVAETEDRK NTLEEYIYTL RGKLEEEYAP FASDAEKTKL
601 QGMLNKAEEW LYDEGFDSIK AKYIAKYEEL ASLGNIIRGR YLAKEEEKKQ
651 AIRSKQEASQ MAAMAEKLAA QRKAEAEKKE EKKDTEGDVD MD
```

Spot 6-lower spot (nLC-ESI-MS/MS)

Protein identified: **Hsc82p**

Hsc82

Mowse Score: **136** (ions scores threshold 52)
Nominal mass (M_r): **80850**; Calculated pI value: **4.78**
Peptides matched: **4**
Sequence Coverage: **5%**

Matched peptides shown in **Bold Red**

```
  1 MAGETFEFQA EITQLMSLII NTVYSNKEIF LRELISNASD ALDKIRYQAL
 51 SDPKQLETEP DLFIRITPKP EEKVLEIRDS GIGMTKAELI NNLGTIAKSG
101 TKAFMEALSA GADVSMIGQF GVGFYSLFLV ADRVQVISKN NEDEQYIWES
151 NAGGSFTVTL DEVNERIGRG TVLRLFLKDD QLEYLEEKRI KEVIKRHSEF
201 VAYPIQLLVT KEVEKEVPIP EEEKKDEEKK DEDDKKPKLE EVDEEEEEKK
251 PKTKKVKEEV QELEELNKTK PLWTRNPSDI TQEEYNAFYK SISNDWEDPL
301 YVKHFSVEGQ LEFRAILFIP KRAPFDLFES KKKKNNIKLY VRRVFITDEA
351 EDLIPEWLSF VKGVVDSEDL PLNLSREMLQ QNKIMKVIRK NIVKKLIEAF
401 NEIAEDSEQF DKFYSAFAKN IKLGVHEDTQ NRAALAKLLR YNSTKSVDEL
451 TSLTDYVTRM PEHQKNIYYI TGESLKAVEK SPFLDALKAK NFEVLFLTDP
501 IDEYAFTQLK EFEGKTLVDI TKDFELEETD EEKAEREKEI KEYEPLTKAL
551 KDILGDQVEK VVVSYKLLDA PAAIRTGQFG WSANMERIMK AQALRDSSMS
601 SYMSSKKTFE ISPKSPIIKE LKKRVDEGGA QDKTVKDLTN LLFETALLTS
651 GFSLEEPTSF ASRINRLISL GLNIDEDEET ETAPEASTEA PVEEVPADTE
701 MEEVD
```

Spot 6-upper spot (MALDI fingerprint)

Protein identified: **Hsc82p**

Hsc82

Mowse Score: **75** (threshold 72)
Nominal mass (M_r): **80719**; Calculated pI value: **4.78**
Number of mass values matched: **18**
Sequence Coverage: **29%**

Matched peptides shown in **Bold Red**

```
  1 AGETFEFQAE ITQLMSLIIN TVYSNKEIFL RELISNASDA LDKIRYQALS
```

51 DPK**QLETEPD LFIRITPKPE EKVLEIR**DSG IGMTKAELIN NLGTIAKSGT

101 KAFMEALSAG ADVSMIGQFG VGFYSLFLVA DRVQVISKNN EDEQYIWESN

151 AGGSFTVTLD EVNERIGRGT VLR**LFLKDDQ LEYLEEKR**IK EVIKRHSEFV

201 AYPIQLLVTK EVEKEVPIPE EEKKDEEKKD EDDKKPK**LEE VDEEEEEKKP**

251 **K**TKKVKEEVQ ELEELNKTKP LWTRNPSDIT QEEYNAFYKS ISNDWEDPLY

301 VK**HFSVEGQL EFR**AILFIPK **RAPFDLFESK K**KK**NNIKLYV RRVFITDEAE**

351 **DLIPEWLSFV KGVVDSEDLP LNLSR**EMLQQ NKIMKVIRKN IVKKLIEAFN

401 EIAEDSEQFD KFYSAFAKNI K**LGVHEDTQN R**AALAKLLRY NSTK**SVDELT**

451 **SLTDYVTR**MP EHQKNIYYIT GESLKAVEKS PFLDALKAKN FEVLFLTDPI

501 DEYAFTQLKE FEGKTLVDIT KDFELEETDE EKAEREKEIK EYEPLTKALK

551 DILGDQVEKV VVSYK**LLDAP AAIRTGQFGW SANMERIMKA QALR**DSSMSS

601 YMSSKKTFEI SPKSPIIKEL KKRVDEGGAQ DKTVK**DLTNL LFETALLTSG**

651 **FSLEEPTSFA SR**INRLISLG LNIDEDEETE TAPEASTEAP VEEVPADTEM

701 EEVD

Spot 7 (MALDI fingerprint)

Protein identified: **Eno2p**

Eno2

Mowse Score: **140** (threshold 53)

Nominal mass (M_r): **46914**; Calculated pI value: **5.67**

Number of mass values matched: **19**

Sequence Coverage: **53%**

Matched peptides shown in **Bold Red**

1 MAVSKVYARS VYDSRGNPTV EVELTTEKGV FR**SIVPSGAS TGVHEALEMR**

51 DEDKSKWMGK GVMNAVNNVN NVIAAAFVKA NLDVKDQKAV DDFLLSLDGT

101 ANKSK**LGANA ILGVSMAAAR** AAAAEK**NVPL YQHLADLSK**S K**TSPYVLPVP**

151 **FLNVLNGGSH AGGALALQEF MIAPTGAK**TF AEAMRIGSEV YHNLKSLTKK

201 R**YGASAGNVG DEGGVAPNIQ TAEEALDLIV DAIK**AAGHDG KVK**IGLDCAS**

251 **SEFFK**DGKYD LDFKNPESDK SKWLTGVELA DMYHSLMK**RY PIVSIEDPFA**

301 **EDDWEAWSHF FKTAGIQIVA DDLTVTNPAR** IATAIEKKAA DALLLK**VNQI**

351 **GTLSESIKAA QDSFAANWGV MVSHRSGETE DTFIADLVVG LR**TGQIKTGA

401 PARSERLAKL NQLLRIEEEL GDK**AVYAGEN FHHGDKL**

Spot 8 (nLC-ESI-MS/MS)

Protein identified: **Sgt2p**

Sgt2

Mowse Score: **632** (ions scores threshold 52)

Nominal mass (M_r): **37195**; Calculated pI value: **4.68**

Peptides matched: **19**

Sequence Coverage: **56%**

Matched peptides shown in **Bold Red**

1 MSASKEEIAA LIVNYFSSIV EKKEISEDGA DSLNVAMDCI SEAFGFEREA
51 VSGILGKSEF KGQHLADILN SASRVPESNK **KDDAENVEIN IPEDDAETK**A
101 KAEDLKMQGN K**AMANKDYEL AINKYTEAIK** VLPTNAIYYA NR**AAAHSSLK**
151 **EYDQAVKDAE SAISIDPSYF R**GYSRLGFAK YAQGKPEEAL EAYKK**VLDIE**
201 **GDNATEAMKR** DYESAKKKVE QSLNLEKTVP EQSR**DADVDA SQGASAGGLP**
251 **DLGSLLGGGL GGLMNNPQLM QAAQKMMSNP GAMQNIQKMM QDPSIRQMAE**
301 **GFASGGGTPN LSDLMNNPAL RNMAGNLFGG AGAQSTDETP DNENKQ**

Spot 9-left spot (MALDI fingerprint)

Protein identified: **Sti1p**

Sti1

Mowse Score: **113** (threshold 63)
Nominal mass (M_r): **66224**; Calculated pI value: **5.45**
Number of mass values matched: **13**
Sequence Coverage: **27%**

Matched peptides shown in **Bold Red**

1 MSLTADEYKQ QGNAAFTAKD YDKAIELFTK **AIEVSETPNH VLYSNR**SACY
51 TSLKKFSDAL NDANECVKIN PSWSKGYNR**L GAAHLGLGDL DEAESNYKK**A
101 LELDASNKAA K**EGLDQVHR**T QQARQAQPDL GLTQLFADPN LIENLKKNPK
151 TSEMMKDPQL VAK**LIGYKQN PQAIGQDLFT DPR**LMTIMAT LMGVDLNMDD
201 INQSNSMPKE PETSKSTEQK KDAEPQSDST TSKENSSKAP QKEESKESEP
251 MEVDEDDSKI EADKEKAEGN KFYKARQFDE AIEHYNK**AWE LHKDITYLNN**
301 **RAAAEYEKGE YETAISTLND AVEQGR**EMRA DYKVISKSFA RIGNAYHKLG
351 DLKKTIEYYQ KSLTEHRTAD ILTKLRNAEK ELKKAEAEAY VNPEKAEEAR
401 LEGKEYFTKS DWPNAVKAYT EMIKRAPEDA RGYSNRAAAL AKLMSFPEAI
451 ADCNK**AIEKD PNFVR**AYIRK ATAQIAVK**EY ASALETLDAA R**TKDAEVNNG
501 SSAR**EIDQLY YK**ASQQR**FQP GTSNETPEET YQR**AMKDPEV AAIMQDPVMQ
551 SILQQAQQNP AALQEHMKNP EVFKK**IQTLI AAGIIR**TGR

Spot 9-right spot (MALDI fingerprint)

Protein identified: **Sti1p**

Sti1

Mowse Score: **93** (threshold 72)
Nominal mass (M_r): **66224**; Calculated pI value: **5.45**
Number of mass values matched: **13**
Sequence Coverage: **29%**

Matched peptides shown in **Bold Red**

1 MSLTADEYKQ QGNAAFTAKD YDKAIELFTK **AIEVSETPNH VLYSNR**SACY
51 TSLKKFSDAL NDANECVKIN PSWSKGYNR**L GAAHLGLGDL DEAESNYKK**A

101 LELDASNKAA KEGLDQVHRT QQARQAQPDL GLTQLFADPN LIENLKKNPK
151 TSEMMKDPQL VAK**LIGYKQN PQAIGQDLFT DPR**LMTIMAT LMGVDLNMDD
201 INQSNSMPKE PETSKSTEQK KDAEPQSDST TSKENSSKAP QKEESKESEP
251 MEVDEDDSKI EADKEKAEGN KFYKARQFDE AIEHYNK**AWE LHKDITYLNN**
301 **RAAAEYEKGE YETAISTLND AVEQGR**EMRA DYKVISKSFA RIGNAYHKLG
351 DLKKTIEYYQ KSLTEHRTAD ILTKLRNAEK ELK**KAEAEAY VNPEKAEEAR**
401 LEGKEYFTKS DWPNAVKAYT EMIKRAPEDA RGYSNRAAAL AKLMSFPEAI
451 ADCNK**AIEKD PNFVR**AYIR**K ATAQIAVKEY ASALETLDAA R**TKDAEVNNG
501 SSAREIDQLY YKASQQR**FQP GTSNETPEET YQR**AMKDPEV AAIMQDPVMQ
551 SILQQAQQNP AALQEHMKNP EVFK**KIQTLI AAGIIR**TGR

Spot 10 (nLC-ESI-MS/MS)

Protein identified: **Hsp104p**

Hsp104

Mowse Score: **1316** (ions scores threshold 52)
Nominal mass (M_r): **101972**; Calculated pI value: **5.31**
Peptides matched: **42**
Sequence Coverage: **36%**

Matched peptides shown in **Bold Red**

1 MNDQTQFTER **ALTILTLAQK** LASDHQHPQL QPIHILAAFI ETPEDGSVPY
51 LQNLIEK**GRY DYDLFK**KVVN RNLVR**IPQQQ PAPAEITPSY ALGK**VLQDAA
101 KIQKQQKDSF IAQDHILFAL FNDSSIQQIF K**EAQVDIEAI KQQALELR**GN
151 TRIDSR**GADT NTPLEYLSKY AIDMTEQAR**Q GKLDPVIGRE EEIRSTIRVL
201 ARRIKSNPCL IGEPGIGKTA IIEGVAQR**II DDDVPTILQG AK**LFSLDLAA
251 LTAGAK**YKGD FEER**FKGVLK EIEESKTLIV LFIDEIHMLM GNGK**DDAANI**
301 **LKPALSR**GQL K**VIGATTNNE YRSIVEKDGA FER**RFQK**IEV AEPSVR**QTVA
351 ILRGLQPK**YE IHHGVRILDS ALVTAAQLAK RYLPYR**RLPD SALDLVDISC
401 AGVAVARDSK PEELDSKERQ LQLIQVEIKA LERDEDADST TKDRLKLARQ
451 K**EASLQEELE PLR**QRYNEEK **HGHEELTQAK** KKLDELENKA LDAERR**YDTA**
501 **TAADLR**YFAI PDIKKQIEK**L EDQVAEEERR AGANSMIQNV VDSDTISETA**
551 **ARLTGIPVK**K LSESENEKLI HMERDLSSEV VGQMDAIKAV SNAVRLSRSG
601 LANPR**QPASF LFLGLSGSGK TELAKKVAGF LFNDEDMMIR** VDCSELSEKY
651 AVSKLLGTTA GYVGYDEGGF LTNQLQYKPY SVLLFDEVEK **AHPDVLTVML**
701 **QMLDDGR**ITS GQGKTIDCSN CIVIMTSNLG AEFINSQQGS KIQESTKNLV
751 MGAVRQHFRP EFLNR**ISSIV IFNK**LSRKAI HKIVDIR**LKE IEER**FEQNDK
801 HYKLNLTQEA KDFLAKYGYS DDMGARPLNR **LIQNEILNK**L ALRILKNEIK
851 **DKETVNVVLK K**GKSRDENVP EEAEECLEVL PNHEATIGAD TLGDDDNEDS
901 MEIDDDLD

Spot 11 (MALDI fingerprint)

Protein identified: **Hsp26p**

Hsp26

Mowse Score: **118** (threshold 53)

Nominal mass (M_r): **23865**; Calculated pI value: **5.31**
Number of mass values matched: **7**
Sequence Coverage: **51%**

Matched peptides shown in **Bold Red**

```
  1 MSFNSPFFDF FDNINNEVDA FNRLLGEGGL RGYAPRRQLA NTPAKDSTGK
 51 EVARPNNYAG ALYDPRDETL DDWFDNDLSL FPSGFGFPRS VAVPVDILDH
101 DNNYELKVVV PGVKSKKDID IEYHQNKNQI LVSGEIPSTL NEESKDKVKV
151 KESSSGKFKR VITLPDYPGV DADNIKADYA NGVLTLTVPK LKPQKDGKNH
201 VKKIEVSSQE SWGN
```

Spot 12-upper spot (MALDI fingerprint)

Protein identified: **Hsp42p**

Hsp42

Mowse Score: **100** (threshold 53)
Nominal mass (M_r): **42848**; Calculated pI value: **4.99**
Number of mass values matched: **8**
Sequence Coverage: **31%**

Matched peptides shown in **Bold Red**

```
  1 MSFYQPSLSL YDVLNALSNQ TGQRGQQGYP RQPQRPQRYH PHYGQVHVGG
 51 HHPRHHPLYS RYNGVPNTYY YQFPGQAYYY SPEYGYDDED GEEEDQDEDM
101 VGDSGTTRQE DGGEDSNSRR YPSYYHCNTA RNNRTNQQAN SLNDLLTALI
151 GVPPYEGTEP EIEANTEQEG EKGEEKDKKD KSEAPKEEAG ETNKEKPLNQ
201 LEESSRPPLA KKSSSFAHLQ APSPIPDPLQ VSKPETRMDL PFSPEVNVYD
251 TEDTYVVVLA LPGANSRAFH IDYHPSSHEM LIKGKIEDRV GIDEKFLKIT
301 ELKYGAFERT VKFPVLPRIK DEEIKATYNN GLLQIKVPKI VNDTEKPKPK
351 KRIAIEEIPD EELEFEENPN PTVEN
```

Spot 12-lower spot (MALDI fingerprint)

Protein identified: **Hsp42p**

Hsp42

Mowse Score: **99** (threshold 53)
Nominal mass (M_r): **42848**; Calculated pI value: **4.99**
Number of mass values matched: **10**
Sequence Coverage: **34%**

Matched peptides shown in **Bold Red**

```
  1 MSFYQPSLSL YDVLNALSNQ TGQRGQQGYP RQPQRPQRYH PHYGQVHVGG
 51 HHPRHHPLYS RYNGVPNTYY YQFPGQAYYY SPEYGYDDED GEEEDQDEDM
```

```
101 VGDSGTTRQE DGGEDSNSRR YPSYYHCNTA RNNRTNQQAN SLNDLLTALI
151 GVPPYEGTEP EIEANTEQEG EKGEEKDKKD KSEAPKEEAG ETNKEKPLNQ
201 LEESSRPPLA KKSSSFAHLQ APSPIPDPLQ VSKPETRMDL PFSPEVNVYD
251 TEDTYVVVLA LPGANSRAFH IDYHPSSHEM LIKGKIEDRV GIDEKFLKIT
301 ELKYGAFERT VKFPVLPRIK DEEIKATYNN GLLQIKVPKI VNDTEKPKPK
351 KRIAIEEIPD EELEFEENPN PTVEN
```

Spot 13 (MALDI fingerprint)

Protein identified: **Bgl2p**

Bgl2

Mowse Score: **69** (threshold 53)
Nominal mass (M_r): **34325**; Calculated pI value: **4.32**
Number of mass values matched: **7**
Sequence Coverage: **29%**

Matched peptides shown in **Bold Red**

```
  1 MRFSTTLATA ATALFFTASQ VSAIGELAFN LGVKNNDGTC KSTSDYETEL
 51 QALKSYTSTV KVYAASDCNT LQNLGPAAEA EGFTIFVGVW PTDDSHYAAE
101 KAALQTYLPK IKESTVAGFL VGSEALYRND LTASQLSDKI NDVRSVVADI
151 SDSDGKSYSG KQVGTVDSWN VLVAGYNSAV IEASDFVMAN AFSYWQGQTM
201 QNASYSFFDD IMQALQVIQS TKGSTDITFW VGETGWPTDG TNFESSYPSV
251 DNAKQFWKEG ICSMRAWGVN VIVFEAFDED WKPNTSGTSD VEKHWGVFTS
301 SDNLKYSLDC DFS
```

Spot 14 (MALDI fingerprint)

Protein identified: **Pep4p**

Pep4

Mowse Score: **93** (threshold 53)
Nominal mass (M_r): **44699**; Calculated pI value: **4.70**
Number of mass values matched: **13**
Sequence Coverage: **37%**

Matched peptides shown in **Bold Red**

```
  1 MFSLKALLPL ALLLVSANQV AAKVHKAKIY KHELSDEMKE VTFEQHLAHL
 51 GQKYLTQFEK ANPEVVFSRE HPFFTEGGHD VPLTNYLNAQ YYTDITLGTP
101 PQNFKVILDT GSSNLWVPSN ECGSLACFLH SKYDHEASSS YKANGTEFAI
151 QYGTGSLEGY ISQDTLSIGD LTIPKQDFAE ATSEPGLTFA FGKFDGILGL
201 GYDTISVDKV VPPFYNAIQQ DLLDEKRFAF YLGDTSKDTE NGGEATFGGI
251 DESKFKGDIT WLPVRRKAYW EVKFEGIGLG DEYAELESHG AAIDTGTSLI
```

301 TLPSGLAEMI NAEIGAK**KGW TGQYTLDCNT R**DNLPDLIFN FNGYNFTIGP
351 YDYTLEVSGS CISAITPMDF PEPVGPLAIV GDAFLR**KYYS IYDLGNNAVG**
401 **LAK**AI

Band 15 (nLC-ESI-MS/MS)

Protein identified: **Tef2p**

Tef2

Mowse Score: **484** (ions scores threshold 35)
Nominal mass (M_r): **50001**; Calculated pI value: **9.14**
Peptides matched: **25**
Sequence Coverage: **43%**

Matched peptides shown in **Bold Red**

1 MGKEK**SHINV VVIGHVDSGK** STTTGHLIYK CGGIDKR**TIE KFEK**EAAELG
51 KGSFK**YAWVL DKLKAER**ERG ITIDIALWKF ETPK**YQVTVI DAPGHR**DFIK
101 NMITGTSQAD CAILIIAGGV GEFEAGISKD GQTREHALLA FTLGVRQLIV
151 AVNKMDSVK**W DESRFQEIVK ETSNFIK**KVG YNPK**TVPFVP ISGWNGDNMI**
201 **EATTNAPWYK** GWEKETKAGV VKGK**TLLEAI DAIEQPSRPT DKPLRLPLQD**
251 **VYKIGGIGTV PVGRVETGVI KPGMVVTFAP AGVTTEVKSV EMHHEQLEQG**
301 **VPGDNVGFNV K**NVSVKEIRR GNVCGDAKND PPKGCASFNA TVIVLNHPGQ
351 ISAGYSPVLD CHTAHIACR**F DELLEK**NDRR SGKKLEDHPK FLK**SGDAALV**
401 **K**FVPSKPMCV EAFSEYPPLG RFAVRDMR**QT VAVGVIK**SVD KTEKAAKVTK
451 AAQKAAKK
